# Supplementary material for: Microwave Assisted Reactions of Fluorescent Pyrrolodiazine Building Blocks
Source: Molecules. 2019 Oct 18;24(20):3760. doi: 10.3390/molecules24203760 (PMC6832281; doi:10.3390/molecules24203760)
Supplement: Supplementary file 1 [file molecules-24-03760-s001.pdf]

Supporting information for:

Microwave assisted reactions of pyrrolodiazine compounds as potential fluorescent biological markers

Costel Moldoveanu [1, *], Dorina Amariucai-Mantu [1], Violeta Mangalagiu [2], Vasilichia Antoci [1], Dan Maftei [1], Ionel Mangalagiu [1] and Gheorghita Zbancioc [1,*]

1 Chemistry Department, Alexandru Ioan Cuza University of Iasi, 11 Carol 1st Bvd, Iasi -700506, Romania; dorina.mantu@uaic.ro (D.A.M.); vasilichia.antoci@uaic.ro (V.A.); dan.maftei@chem.uaic.ro (D.M.); ionelm@uaic.ro (I.M.)

2 Institute of Interdisciplinary Research- CERNESIM Centre, Alexandru Ioan Cuza University of Iasi, 11 Carol I, Iasi, 700506, Romania; violeta.mangalagiu@uaic.ro (V.M.)

* Correspondence: gheorghita.zbancioc@uaic.ro (G.Z.); costel.moldoveanu@uaic.ro (C.M.) Tel.: +40-232-201278

Contents

1. NMR Spectra of the obtained compounds. 2

2. IR Spectra of the obtained compounds. 15

1. NMR Spectra of the obtained compounds.

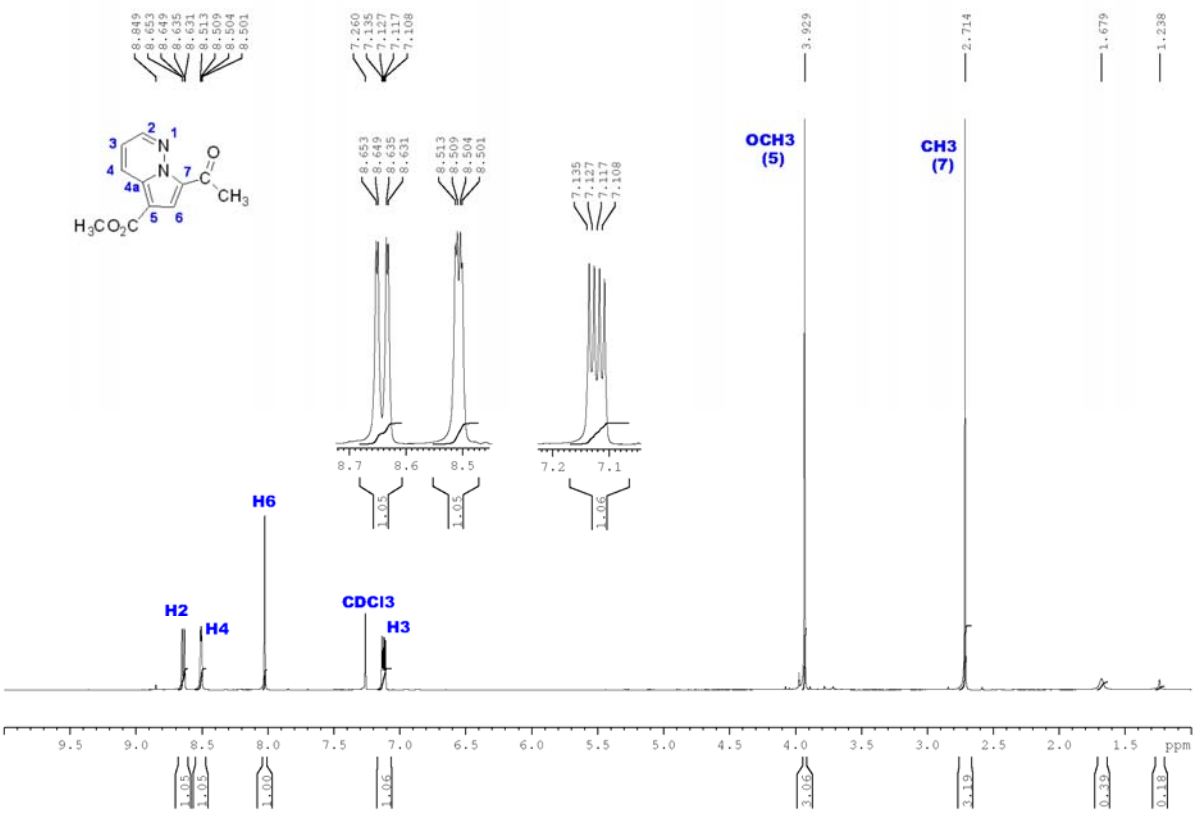

S1a Fig. ^{1}H NMR spectrum of the compound **7.**

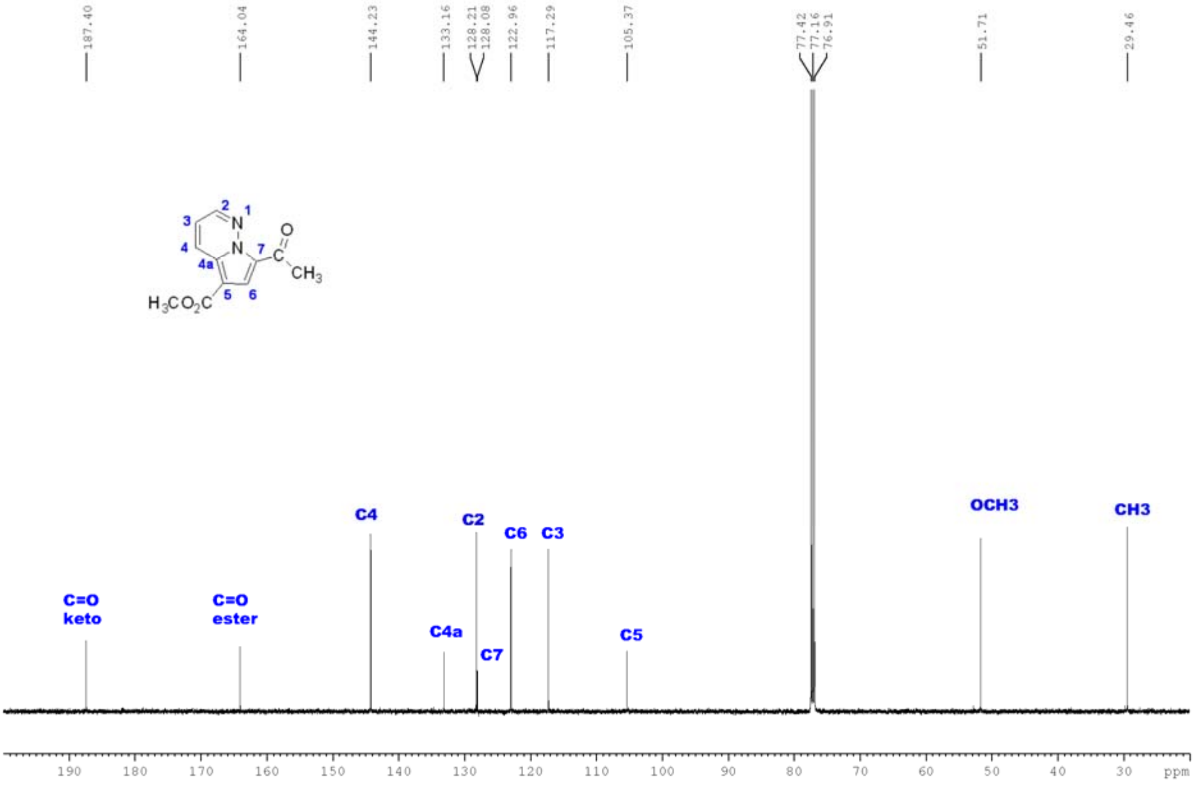

S1b Fig. ^{13}C NMR spectrum of the compound **7.**

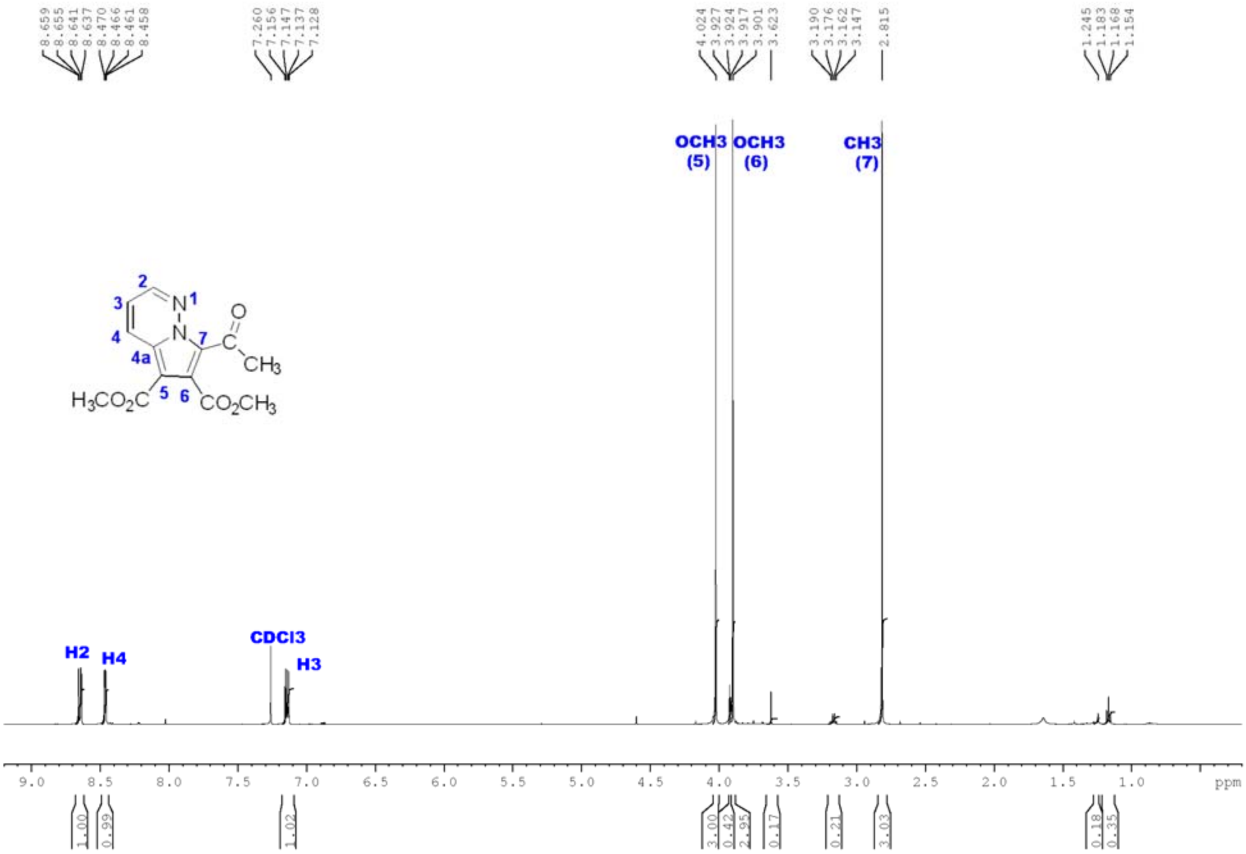

S2a Fig. ¹H NMR spectrum of the compound **8.**

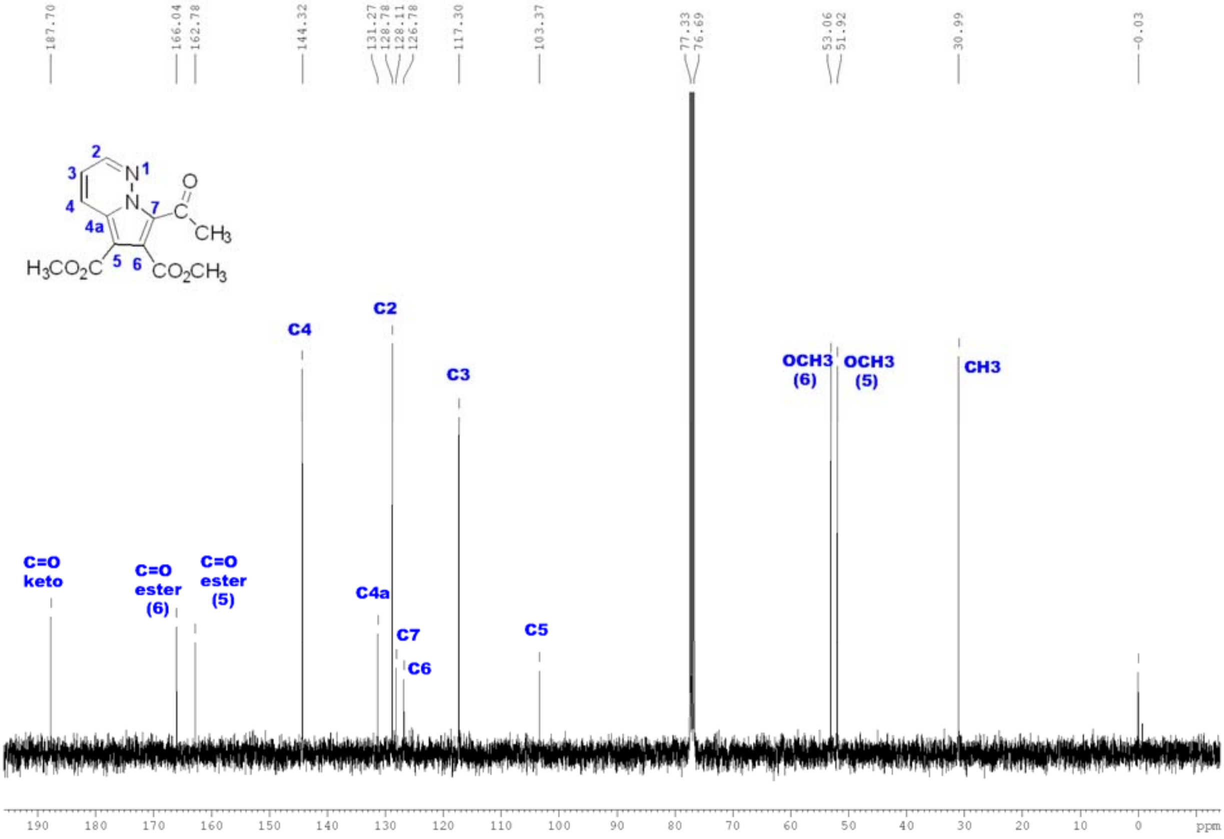

S2b Fig. ¹³C NMR spectrum of the compound **8.**

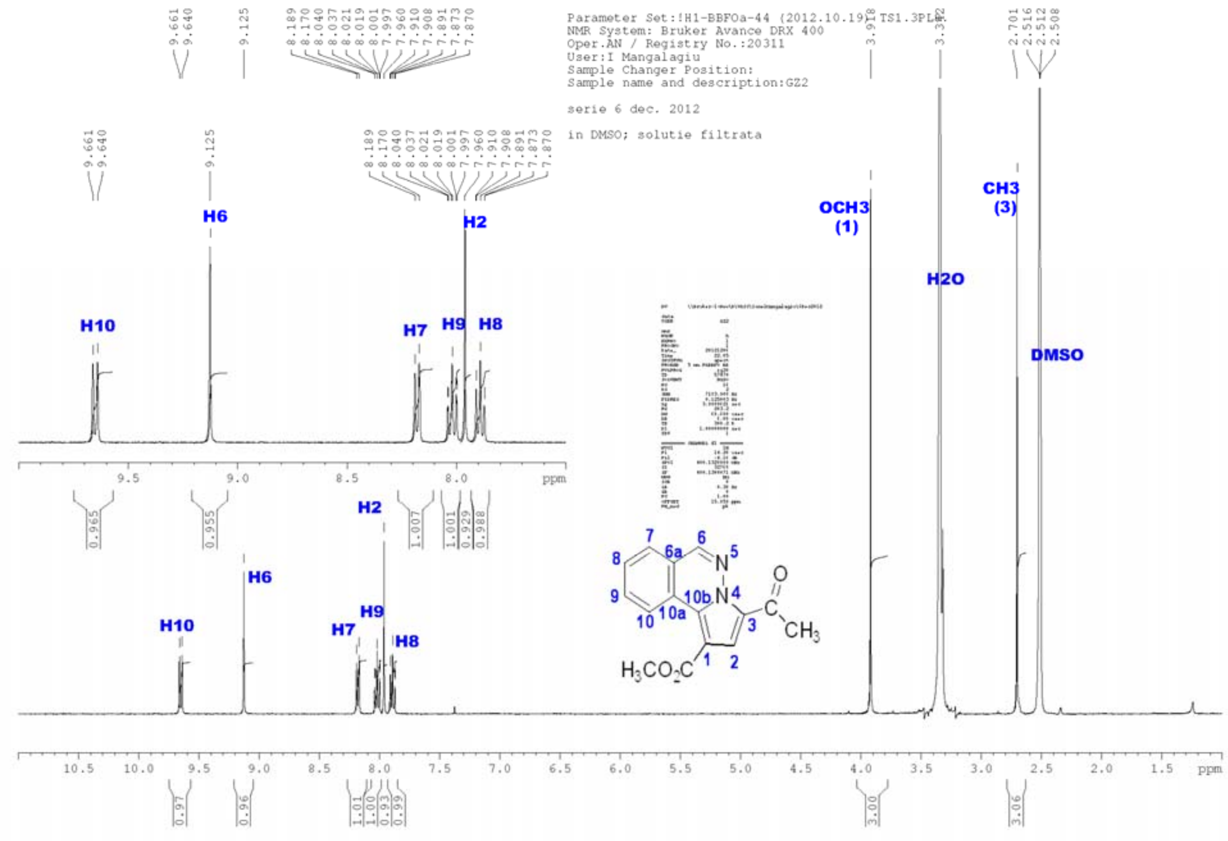

S3a Fig. ^{1}H NMR spectrum of the compound **9.**

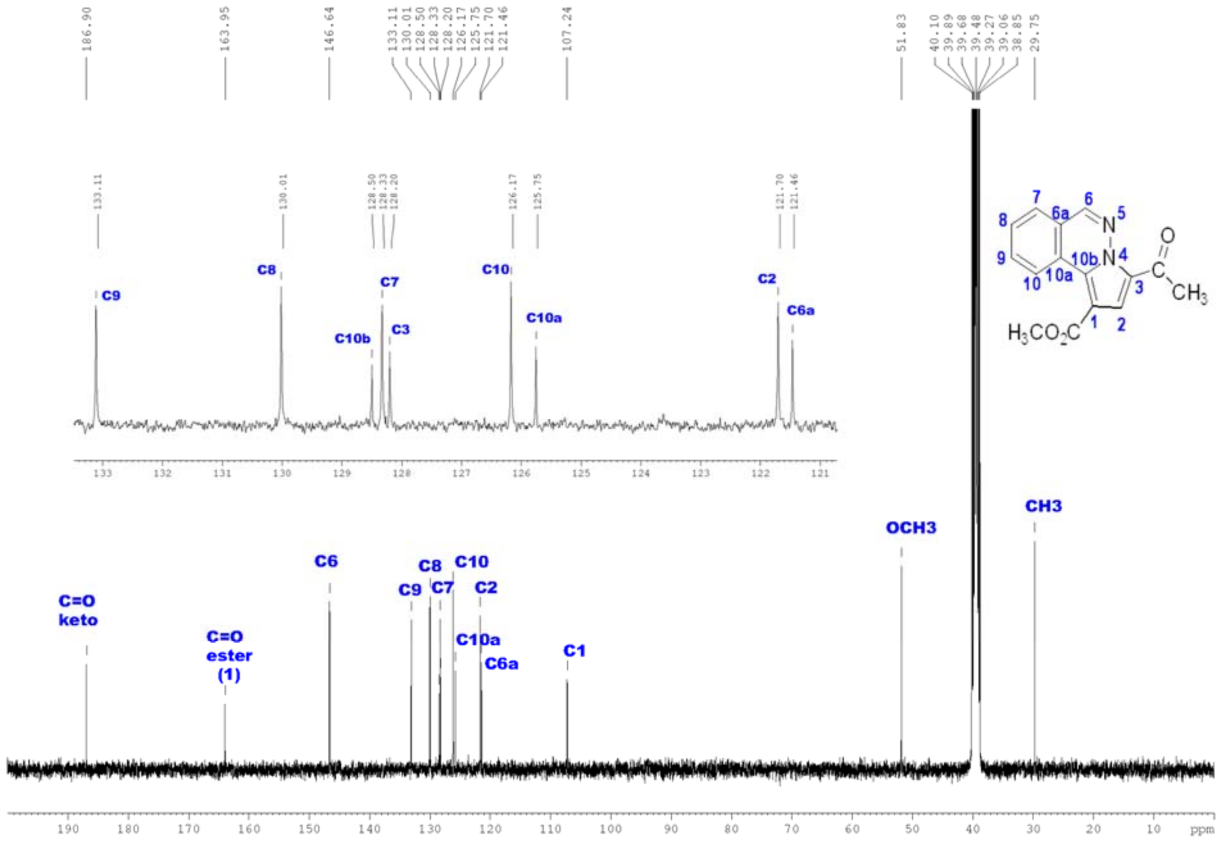

S3b Fig. ^{13}C NMR spectrum of the compound **9.**

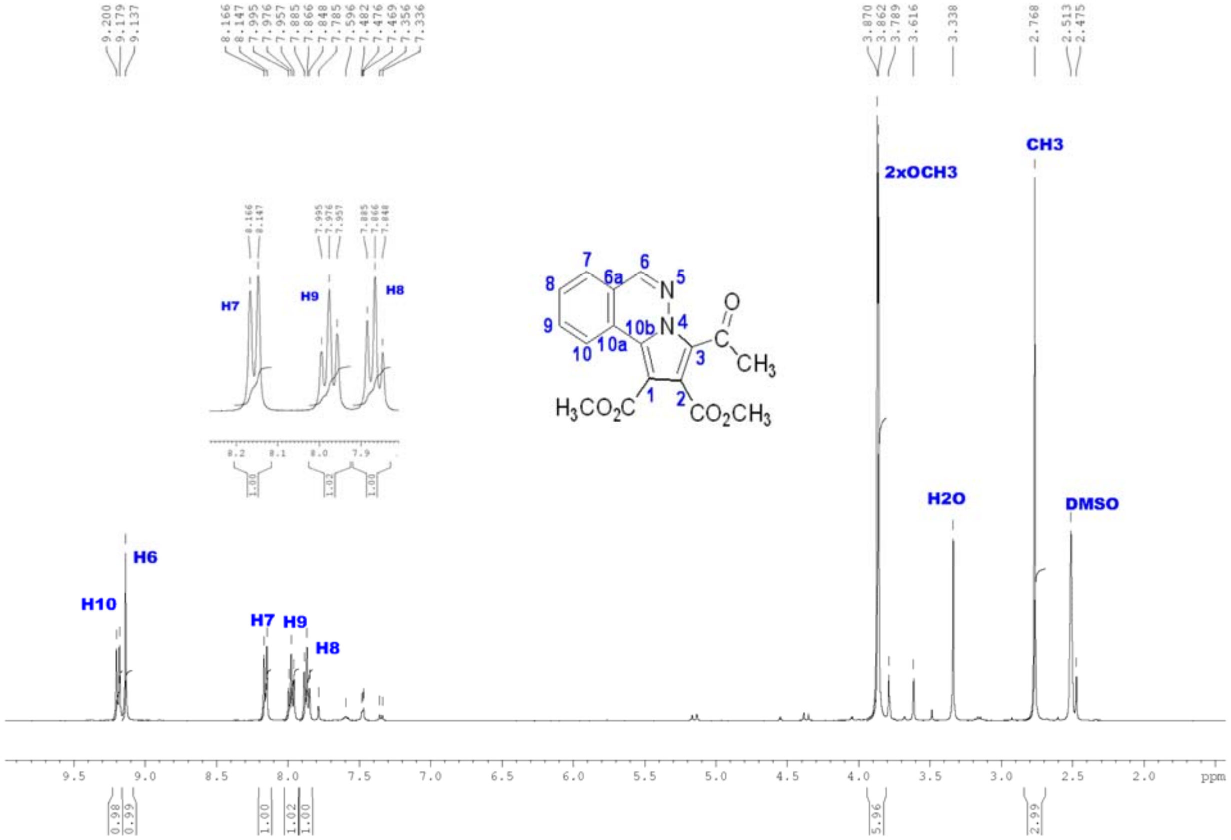

S4a Fig. ^{1}H NMR spectrum of the compound **10.**

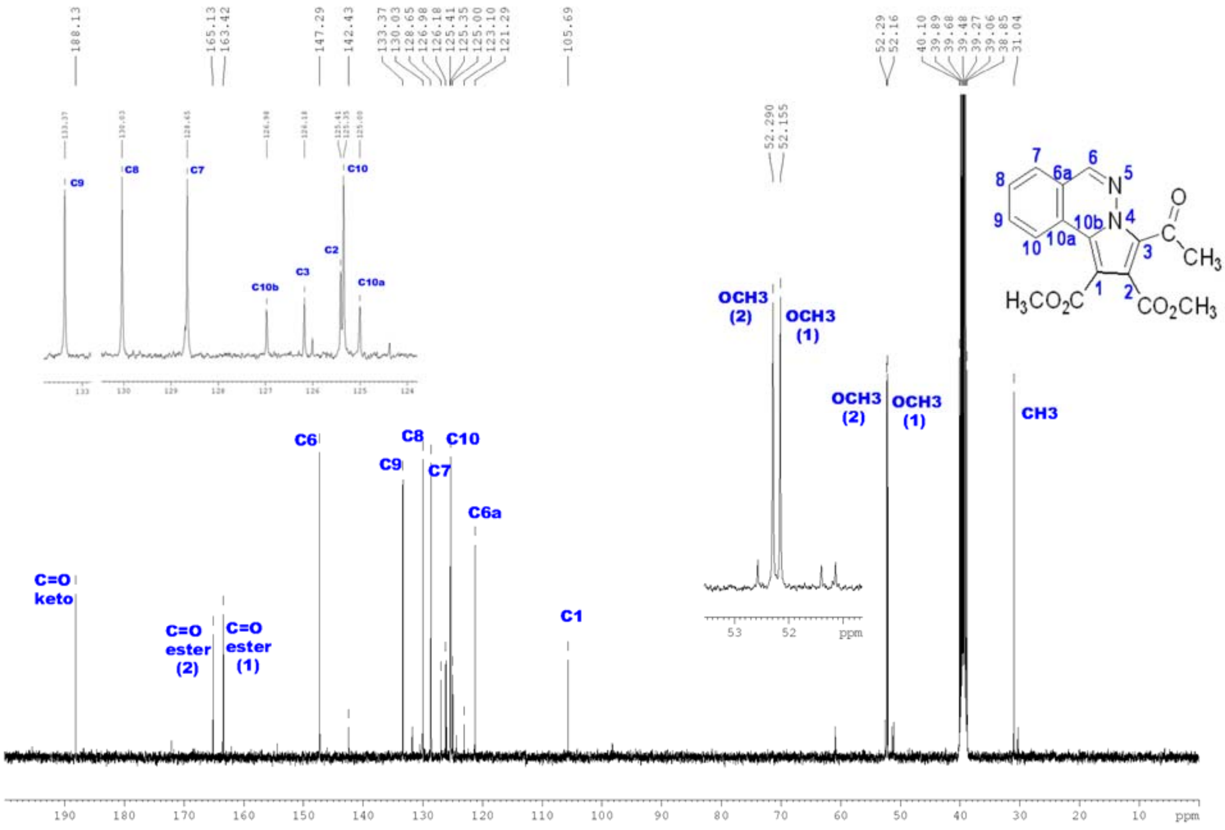

S4b Fig. ^{13}C NMR spectrum of the compound **10.**

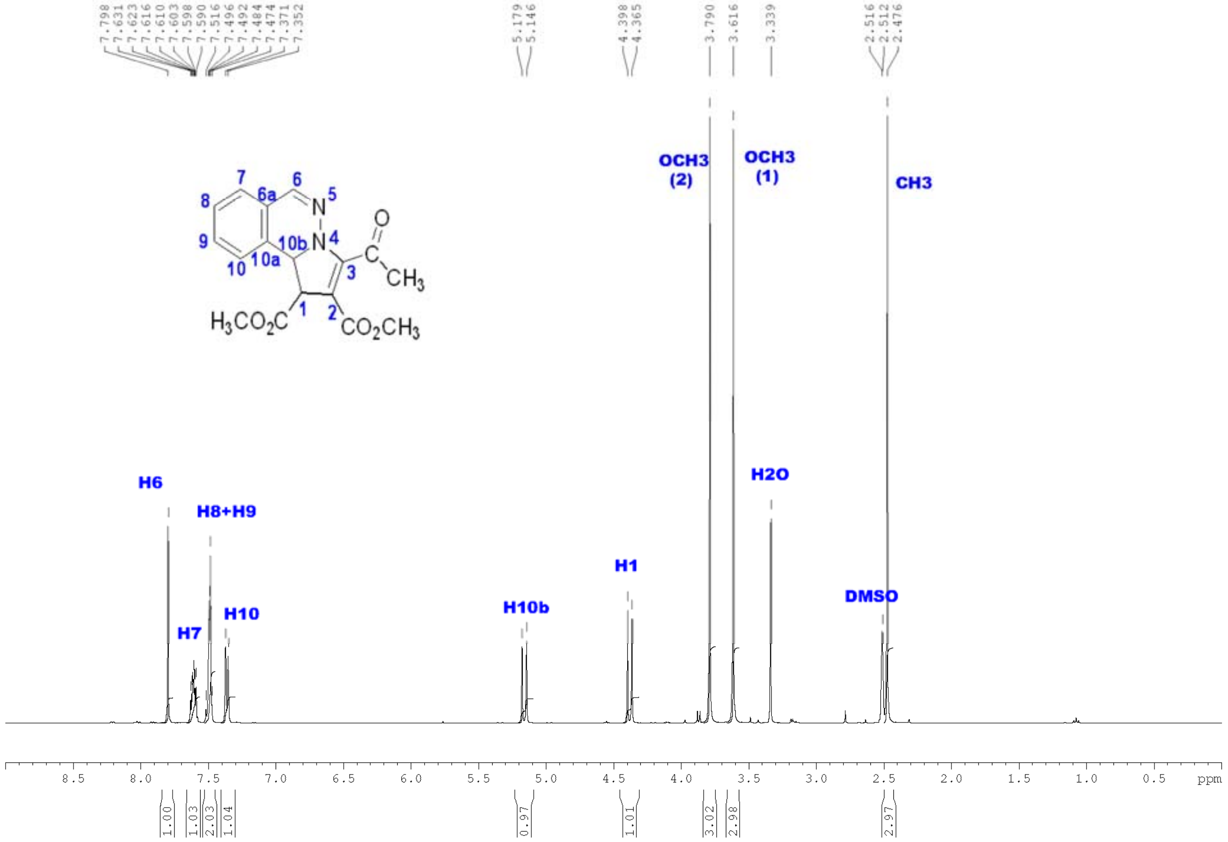

S5a Fig. ^{1}H NMR spectrum of the compound **11.**

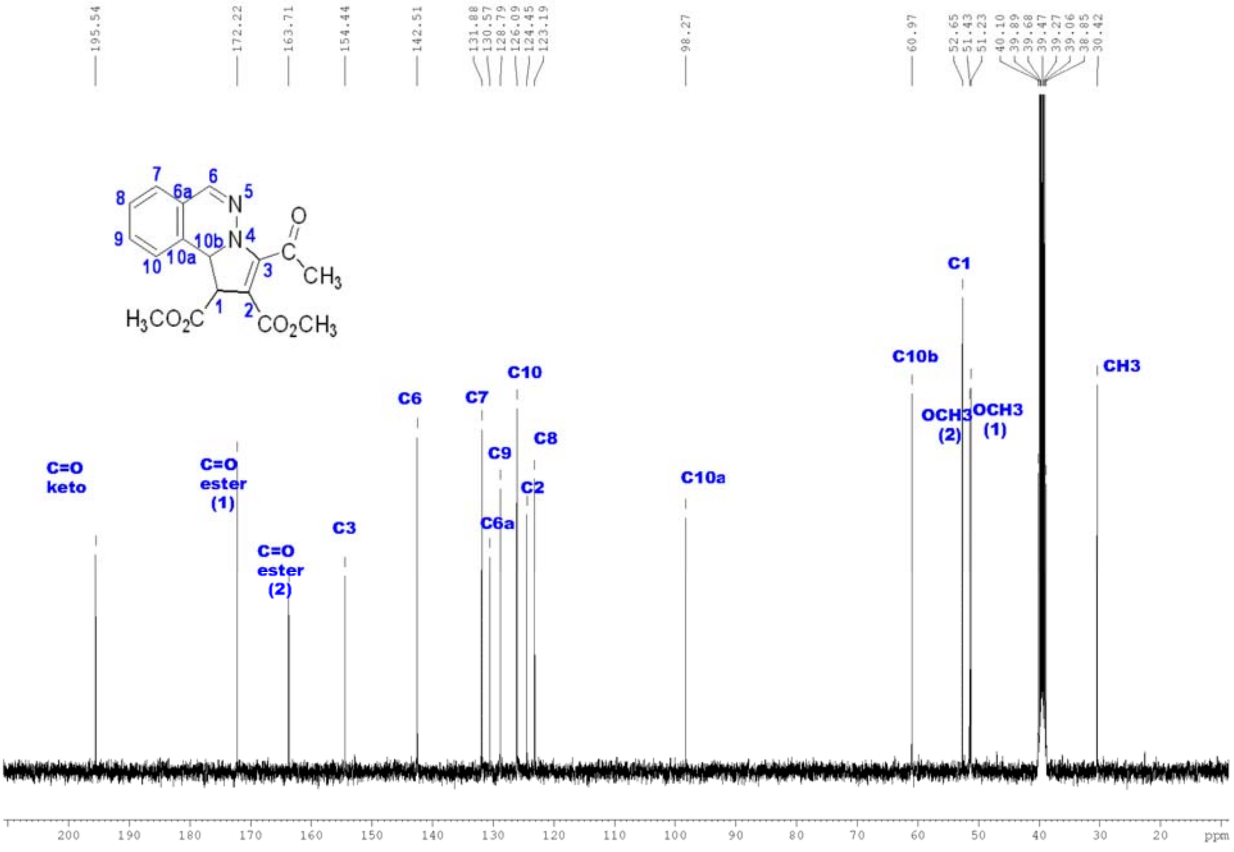

S5b Fig. ^{13}C NMR spectrum of the compound **11.**

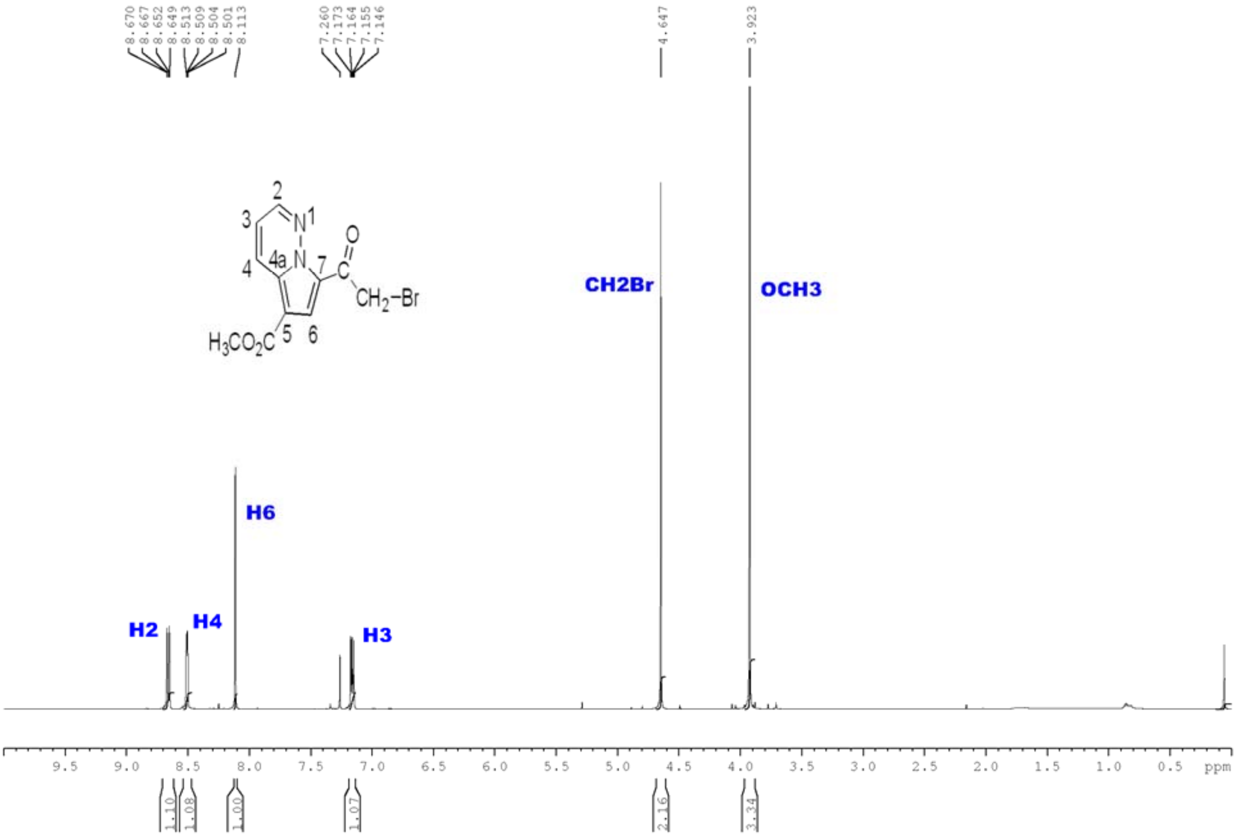

S6a Fig. ^{1}H NMR spectrum of the compound **12a.**

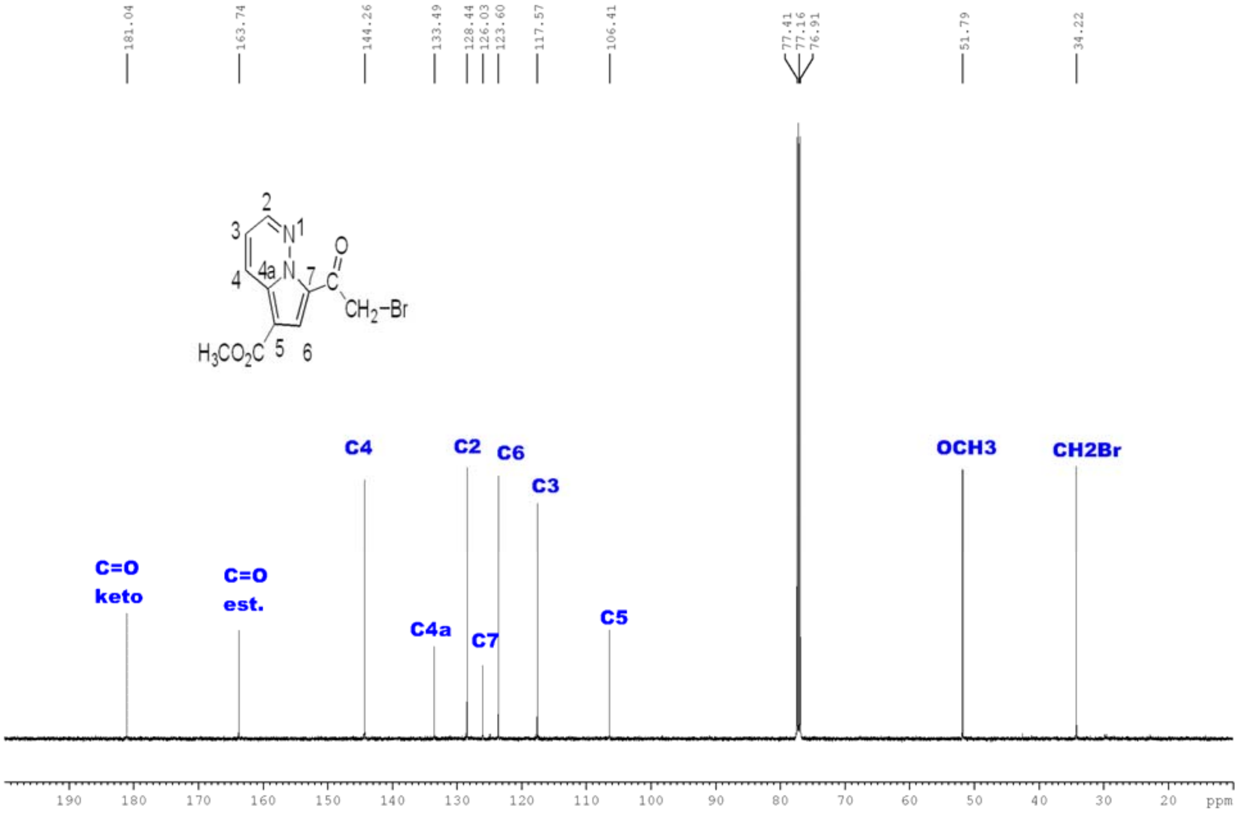

S6b Fig. ^{13}C NMR spectrum of the compound **12a.**

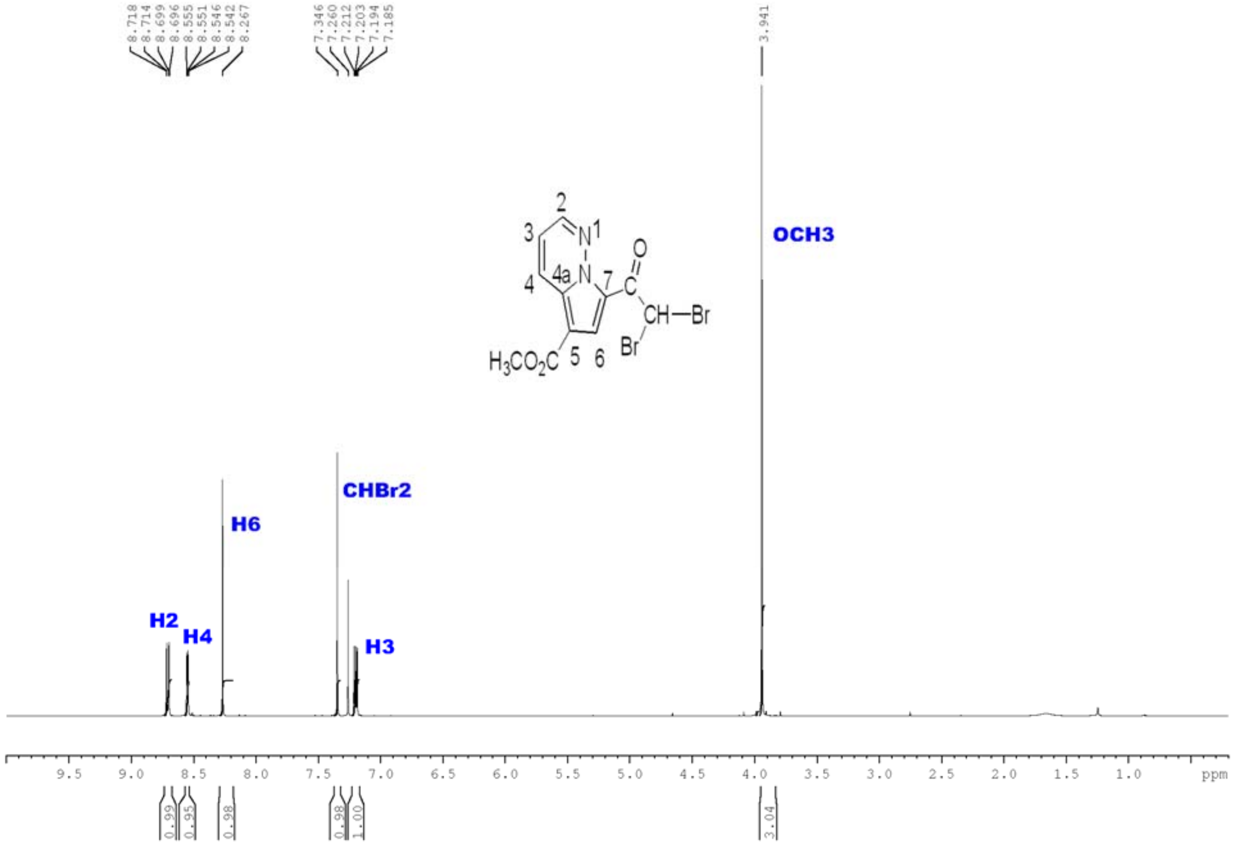

S7a Fig. ^{1}H NMR spectrum of the compound **12b.**

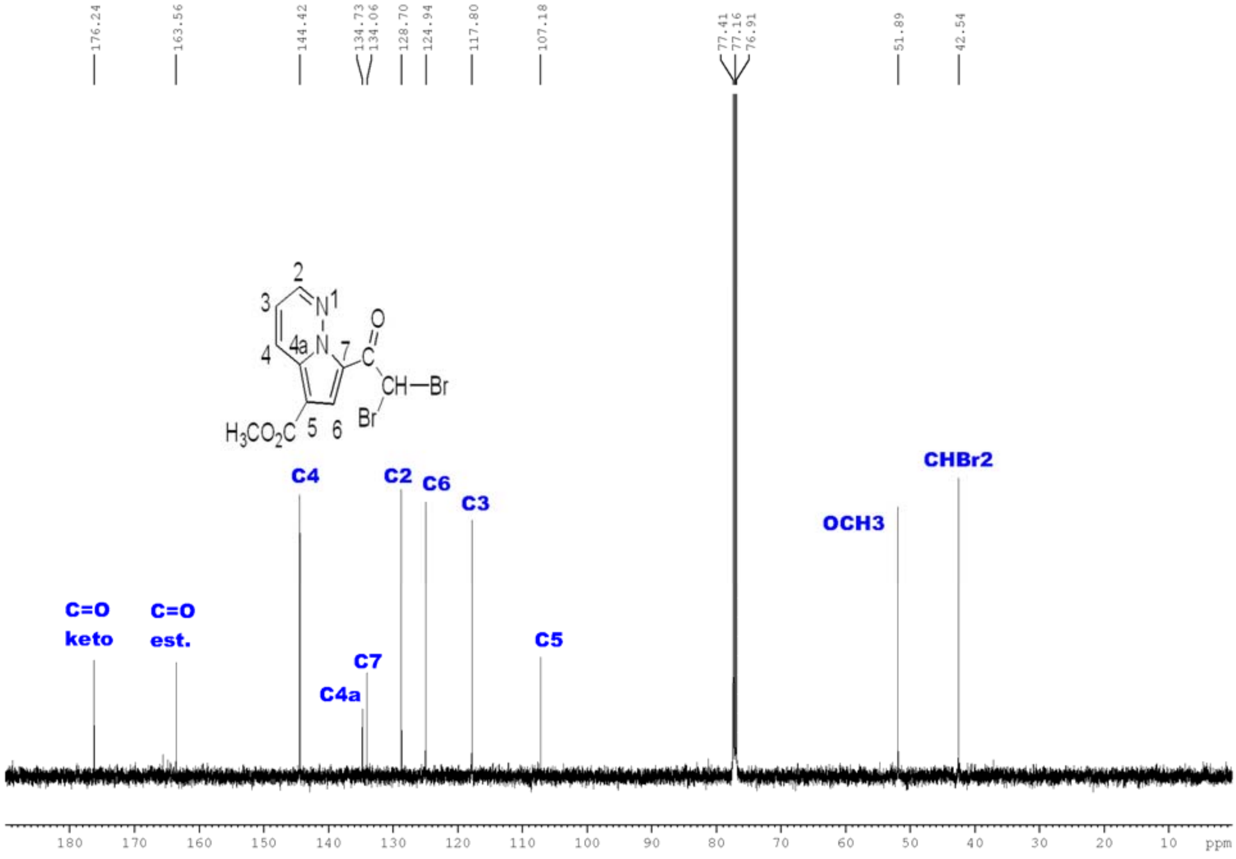

S7b Fig. ^{13}C NMR spectrum of the compound **12b.**

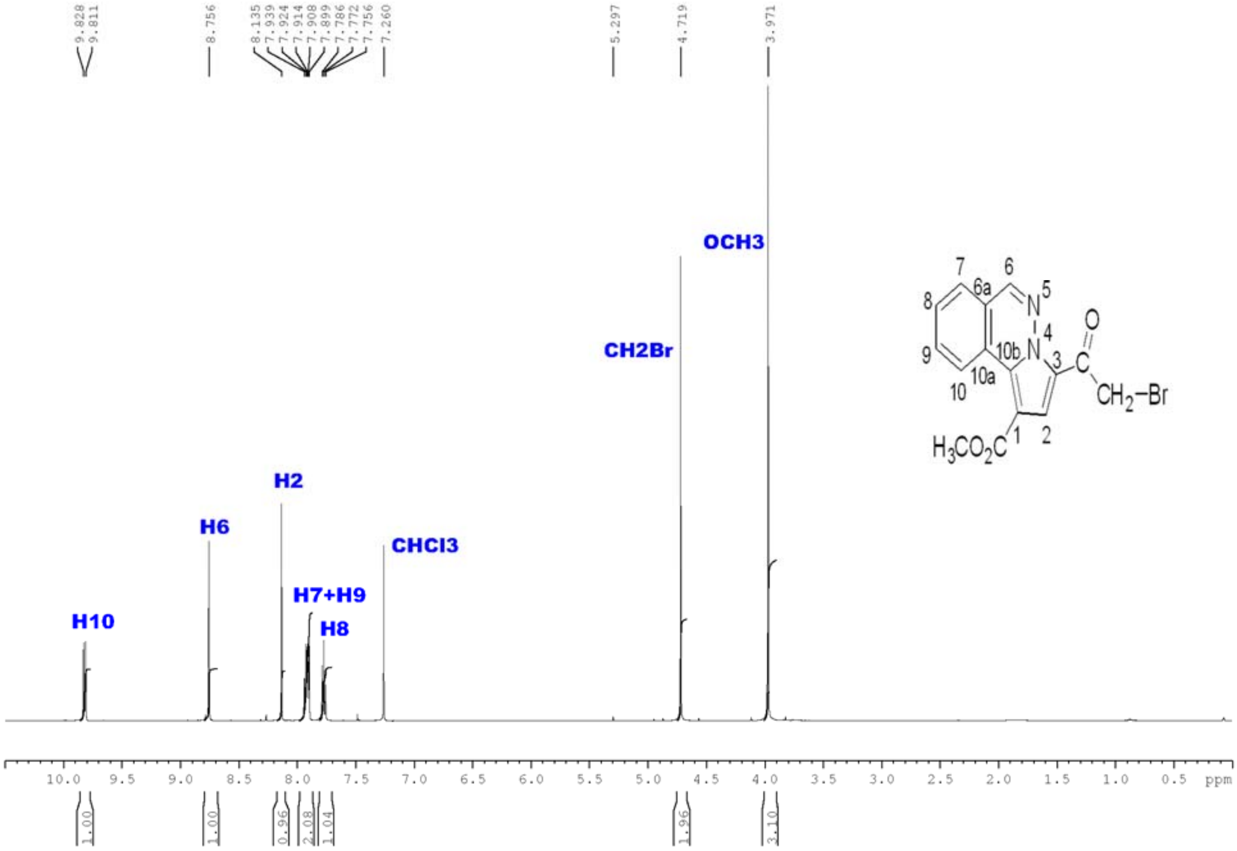

S8a Fig. ^{1}H NMR spectrum of the compound **13a.**

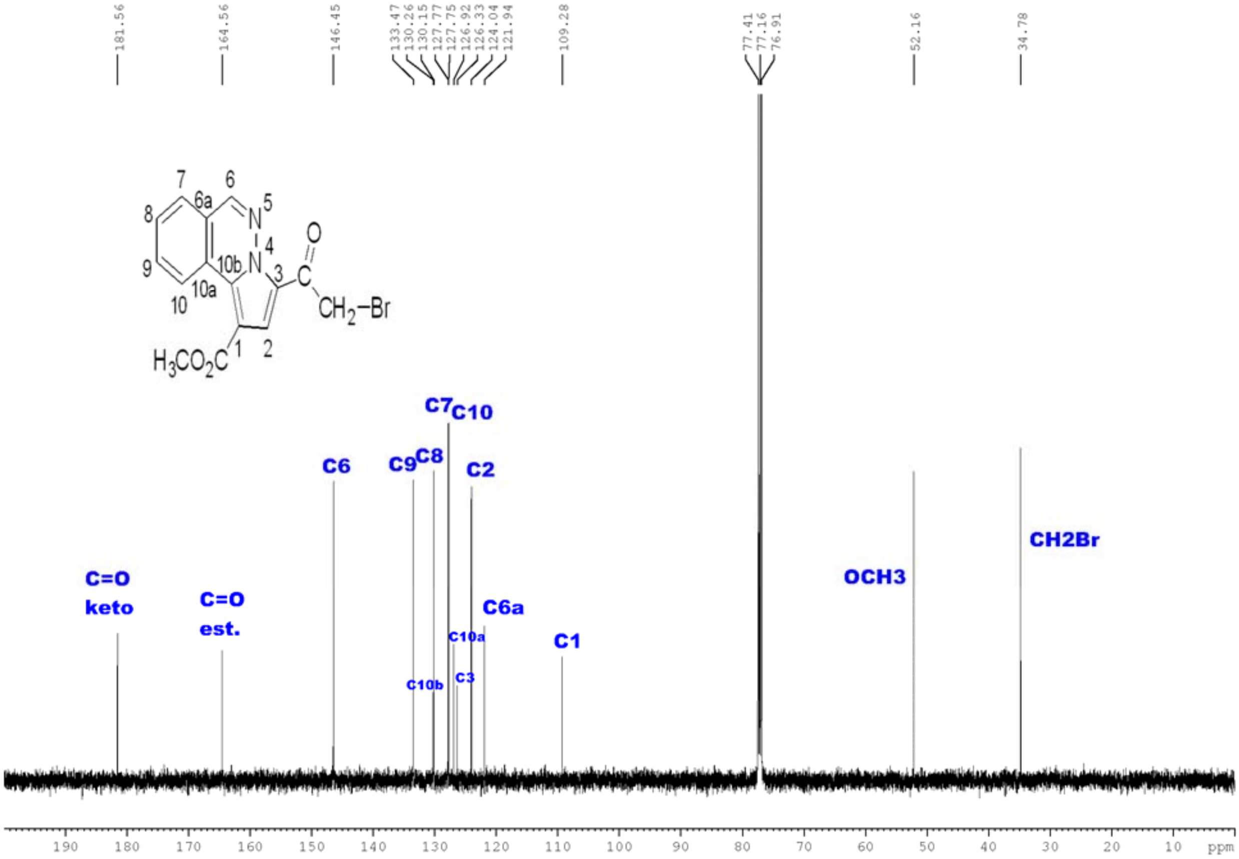

S8b Fig. ^{13}C NMR spectrum of the compound **13a.**

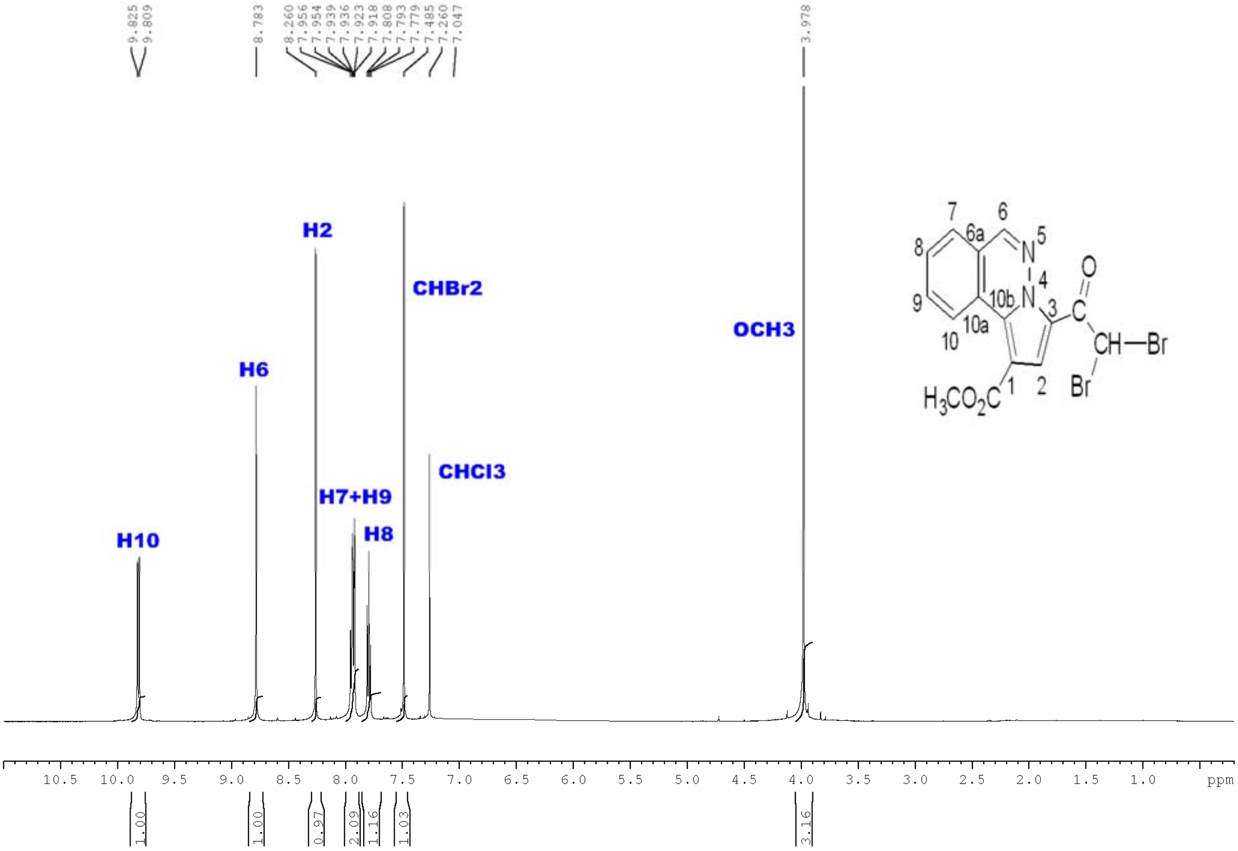

S9a Fig. ^{1}H NMR spectrum of the compound **13b.**

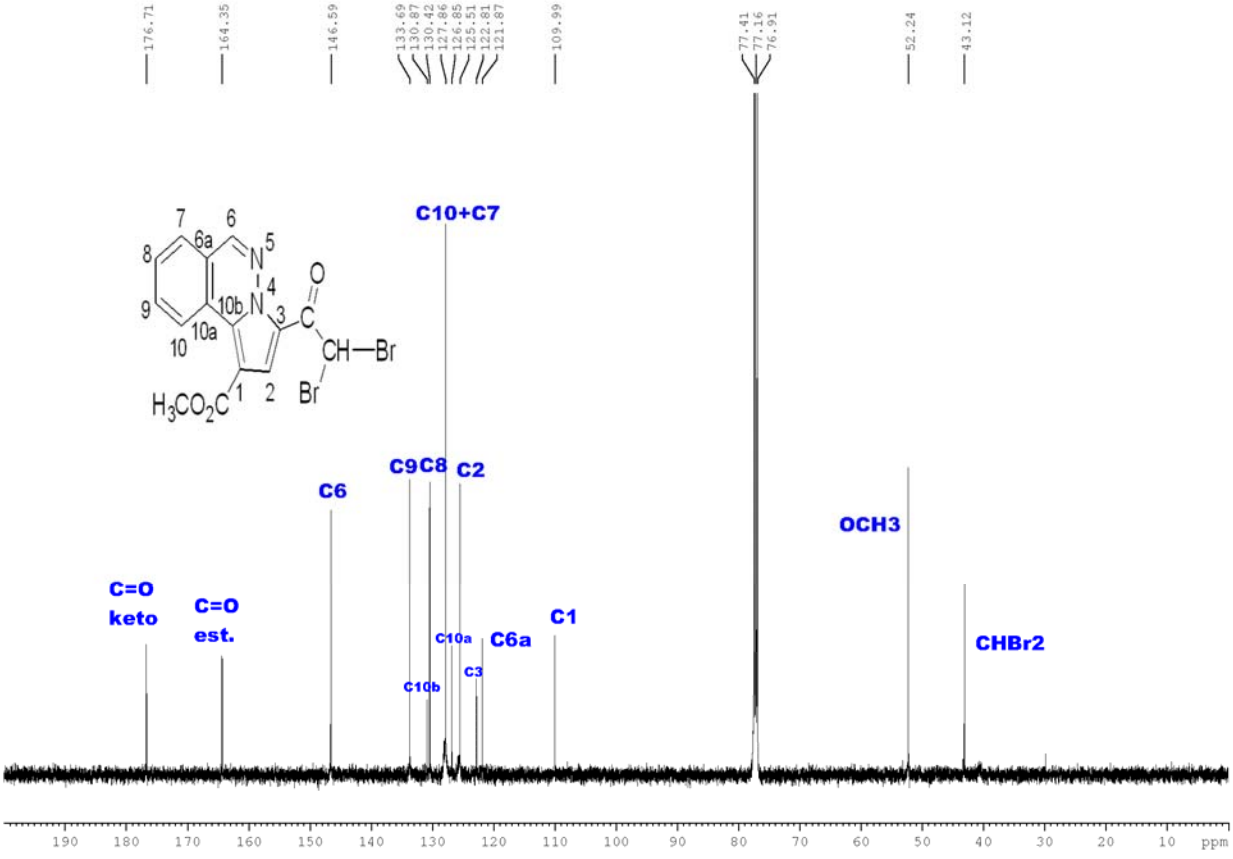

S9b Fig. ^{13}C NMR spectrum of the compound **13b.**

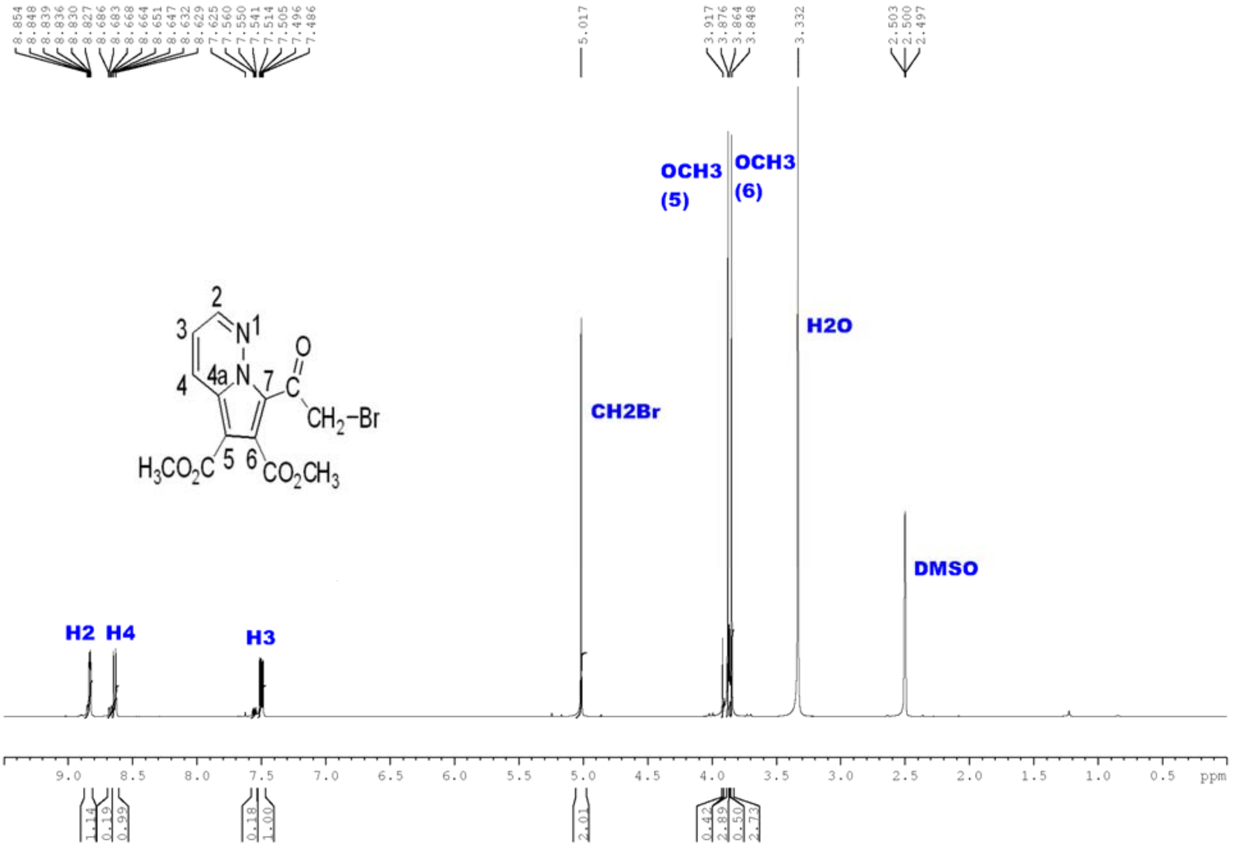

S10a Fig. ^{1}H NMR spectrum of the compound **14a.**

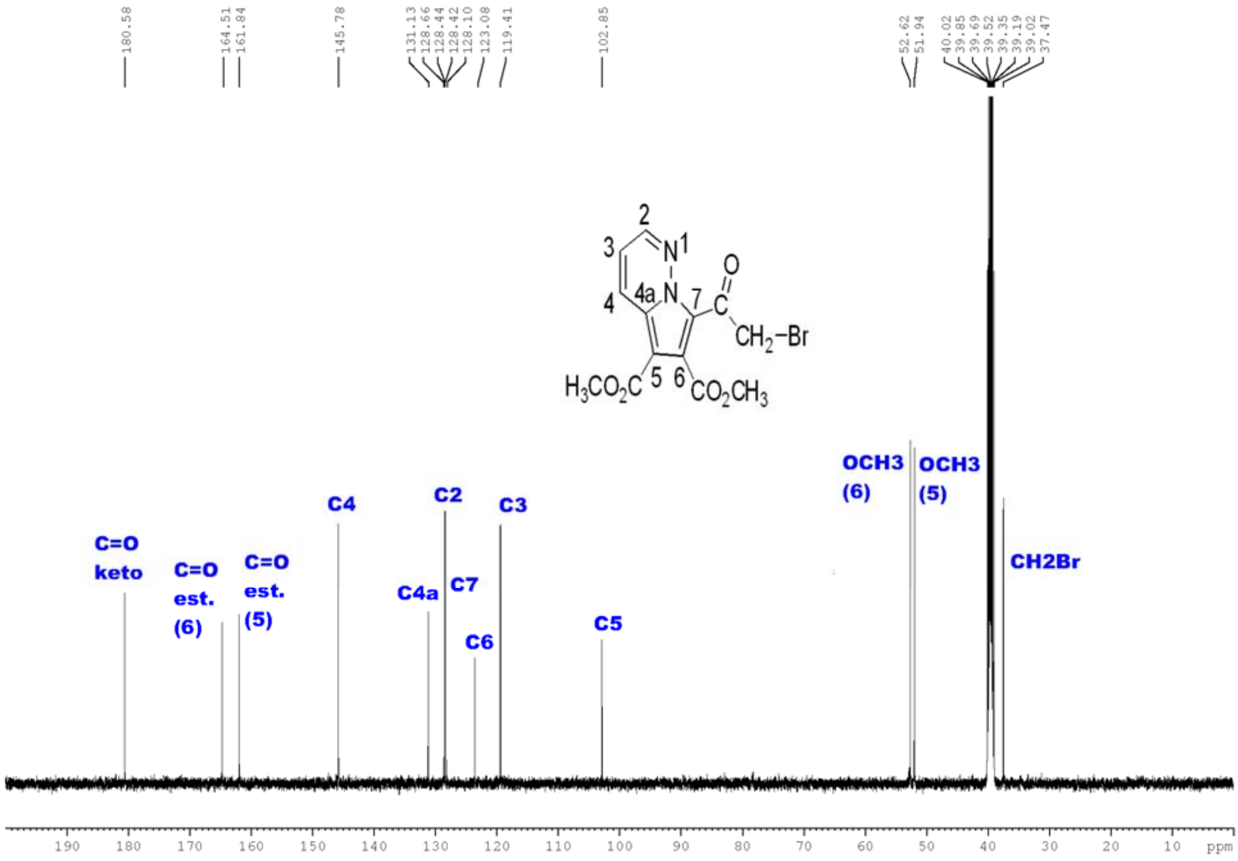

S10b Fig. ^{13}C NMR spectrum of the compound **14a.**

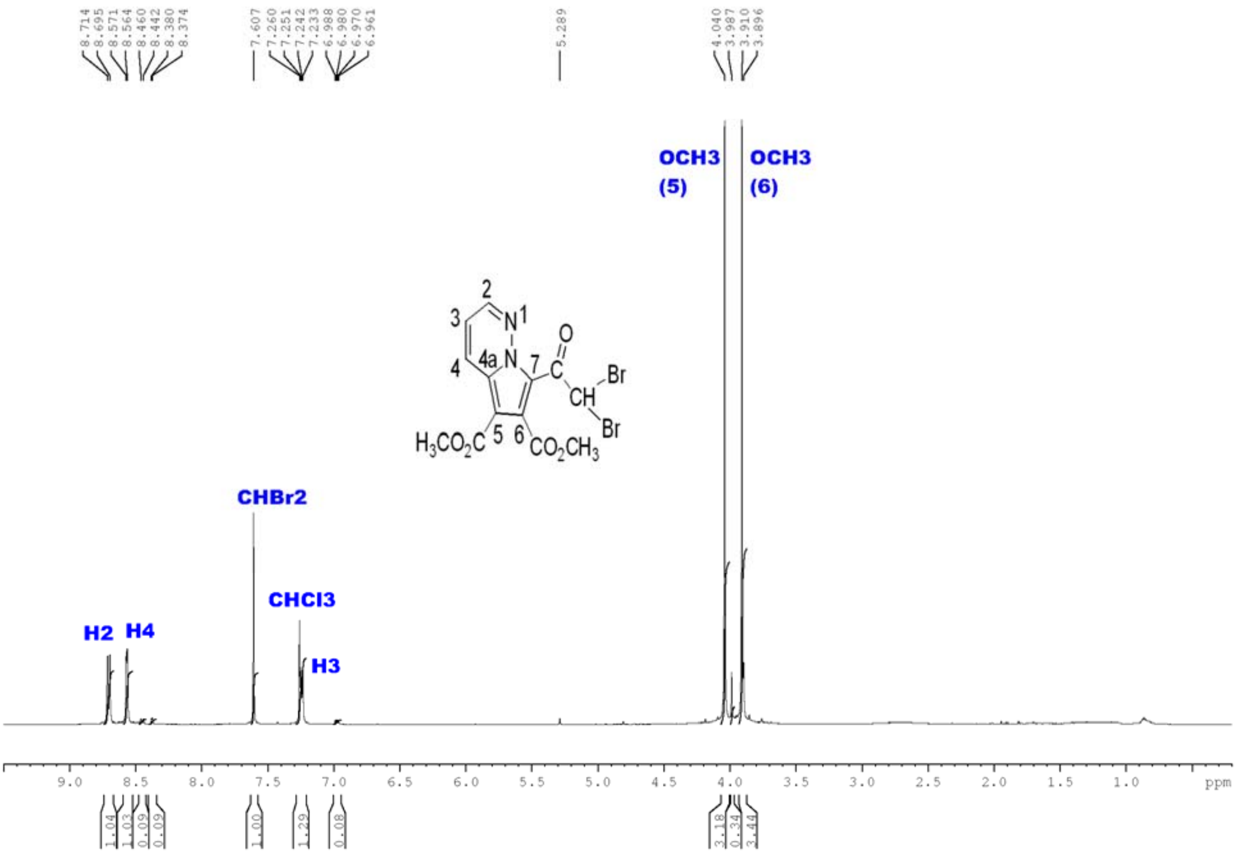

S11a Fig. ^{1}H NMR spectrum of the compound **14b.**

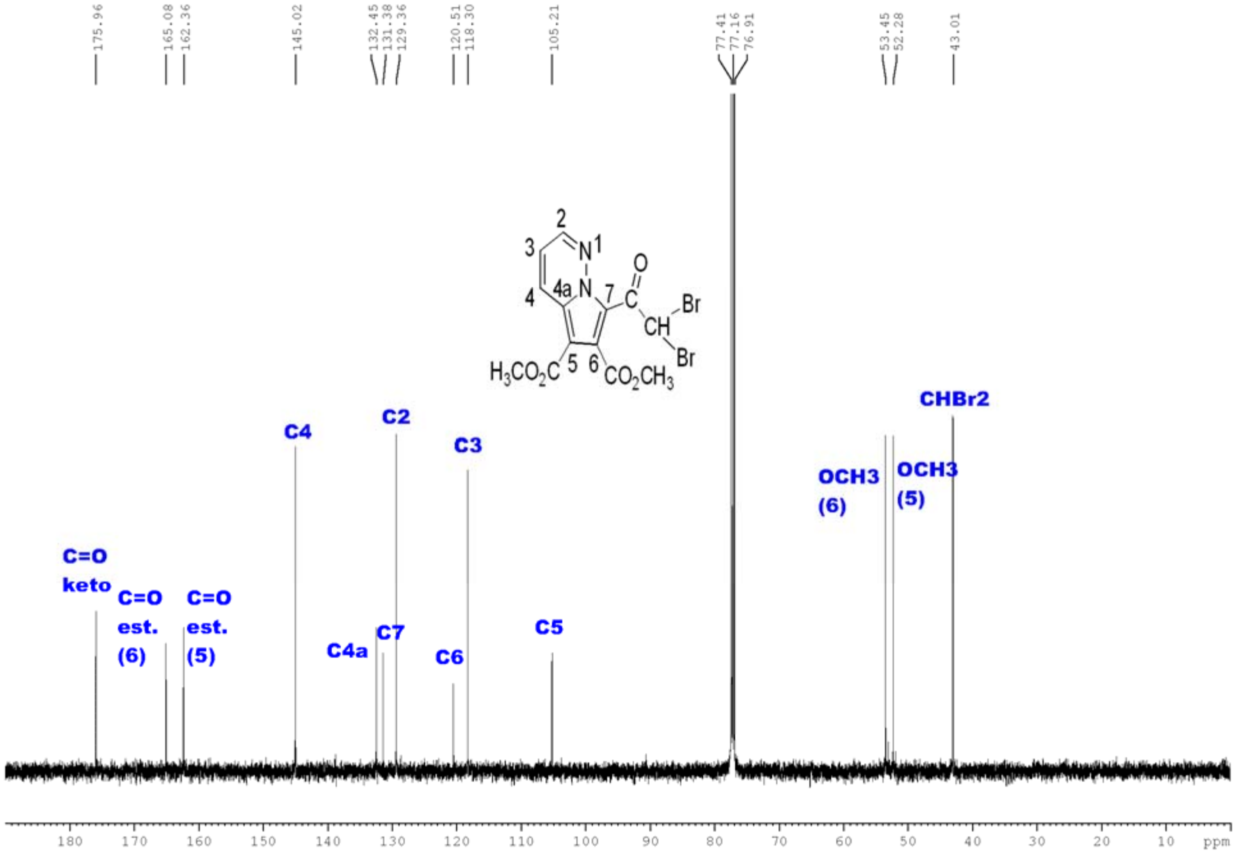

S11b Fig. ^{13}C NMR spectrum of the compound **14b.**

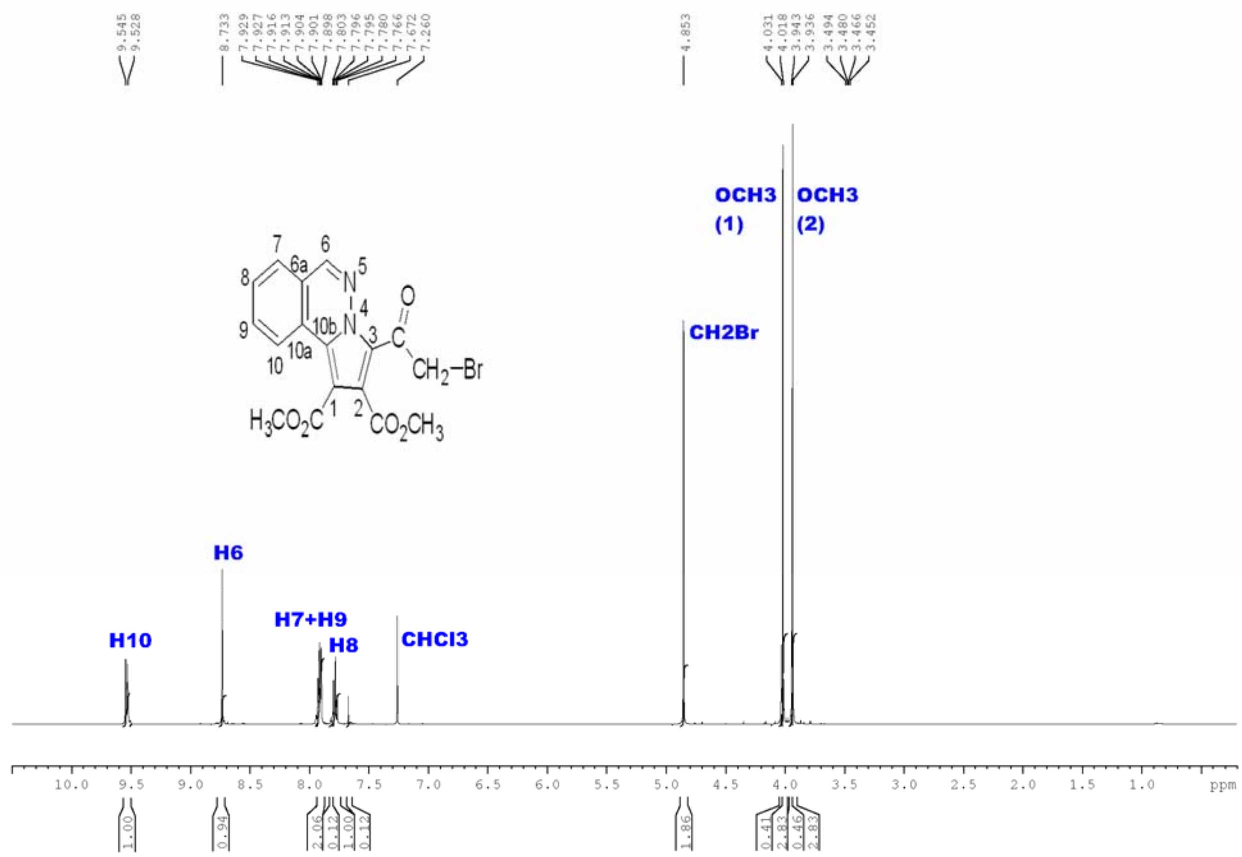

S12a Fig. ^{1}H NMR spectrum of the compound **15a.**

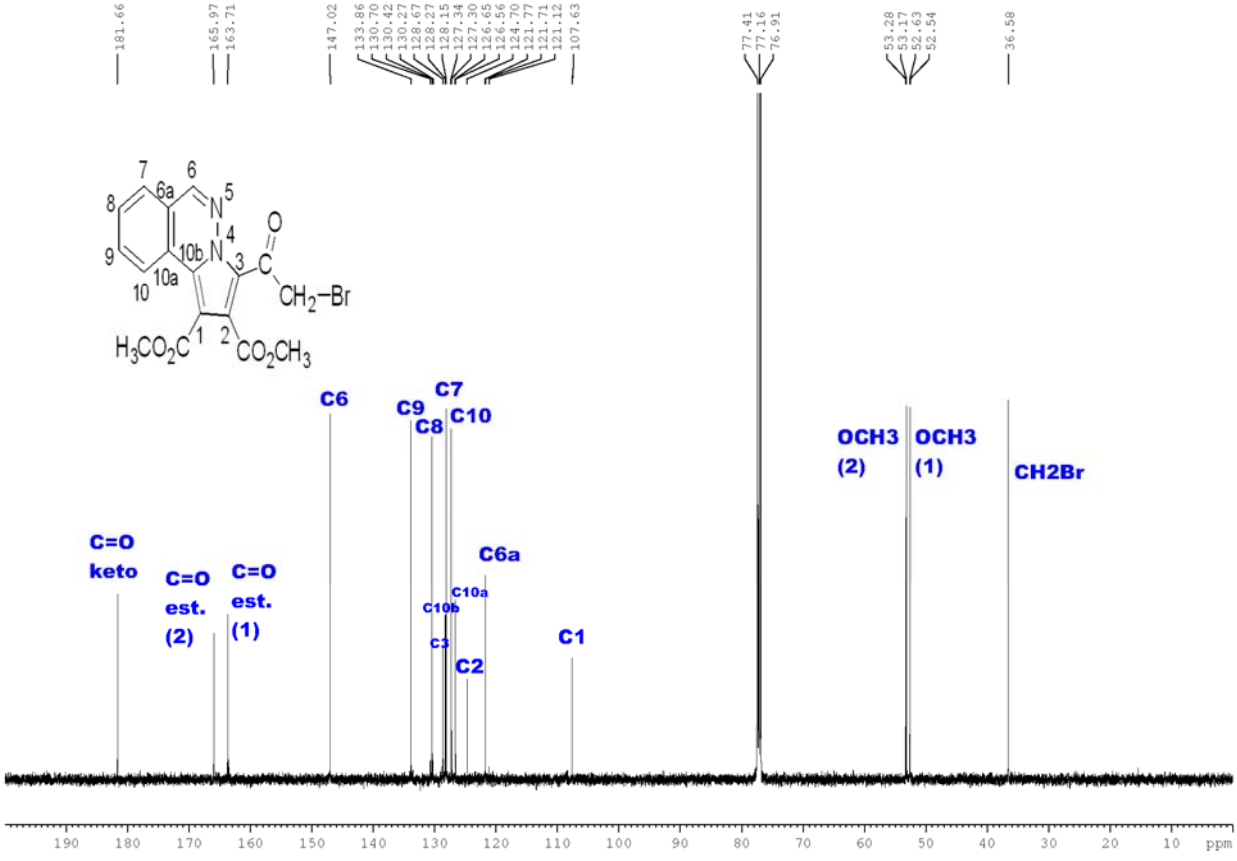

S12b Fig. ^{13}C NMR spectrum of the compound **15a.**

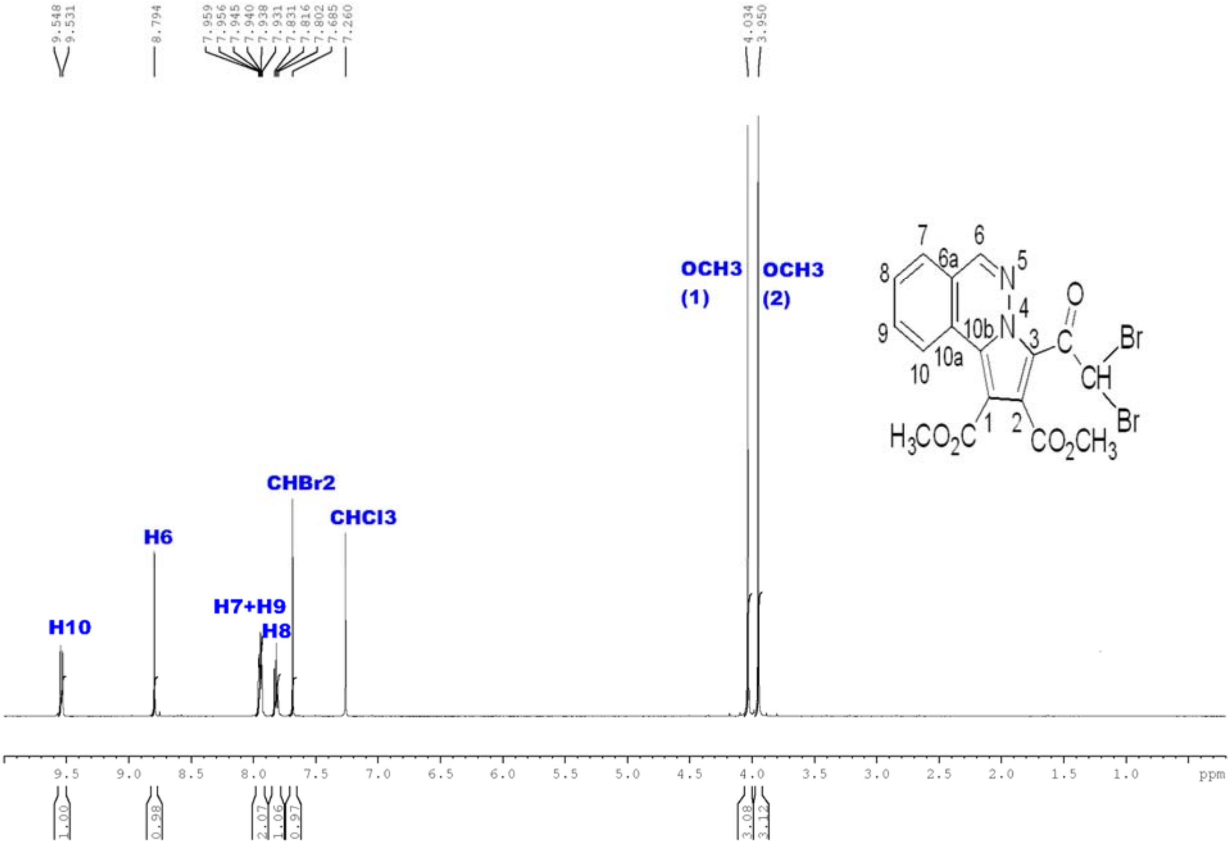

S13a Fig. ^{1}H NMR spectrum of the compound **15b.**

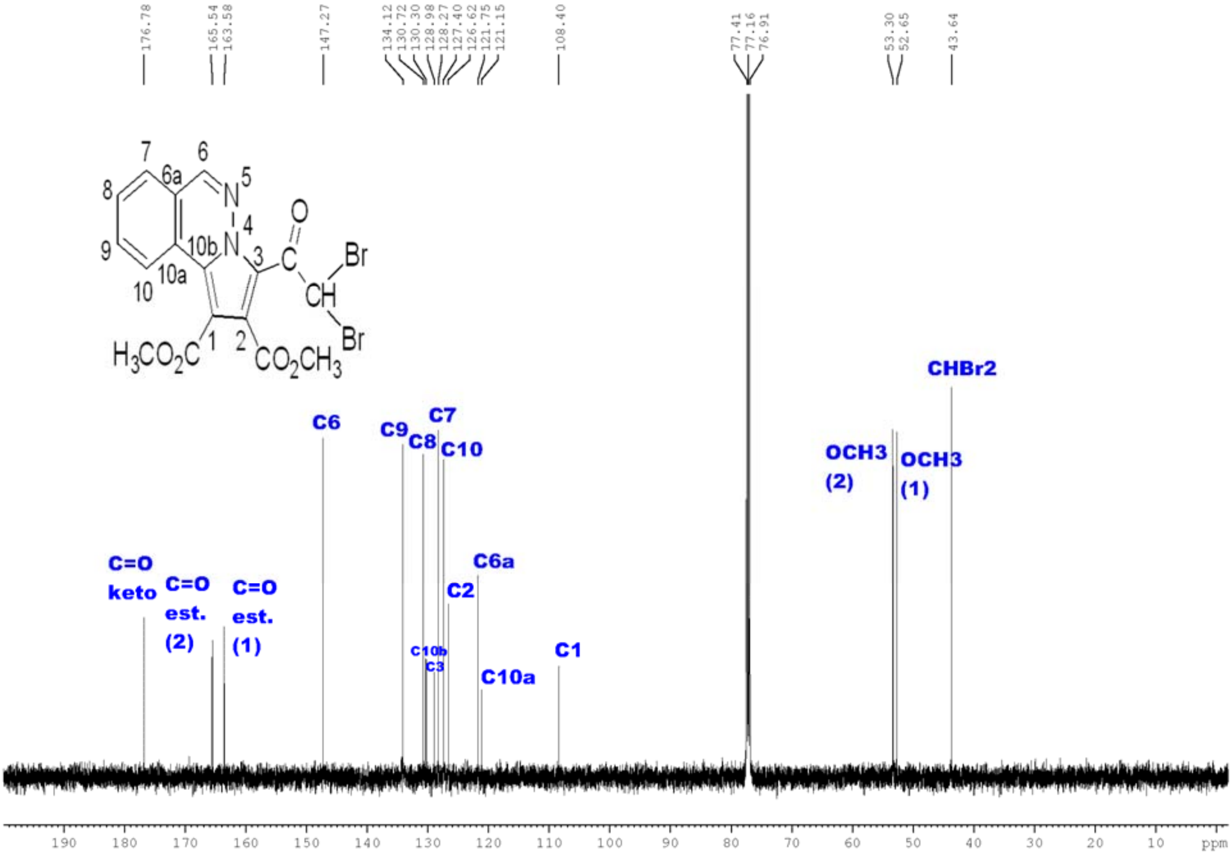

S13b Fig. ^{13}C NMR spectrum of the compound **15b.**

2. IR Spectra of the obtained compounds.

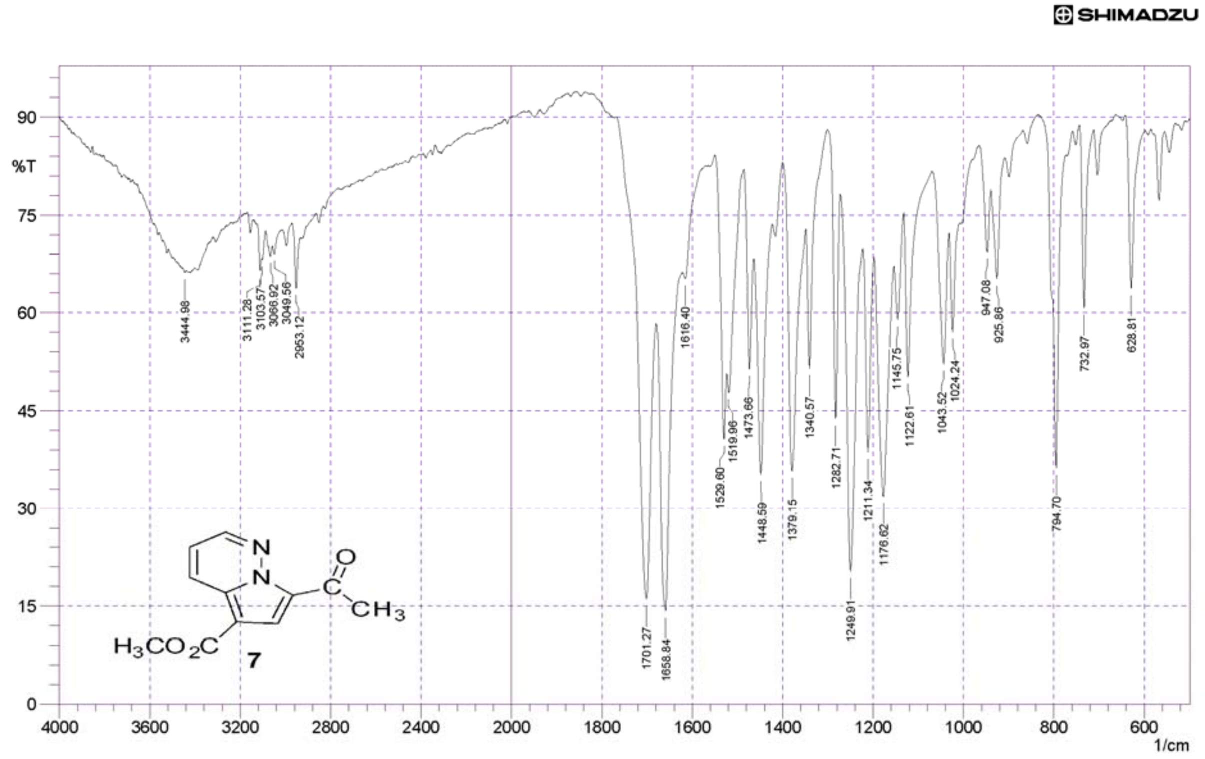

S14 Fig. IR spectrum of the compound **7.**

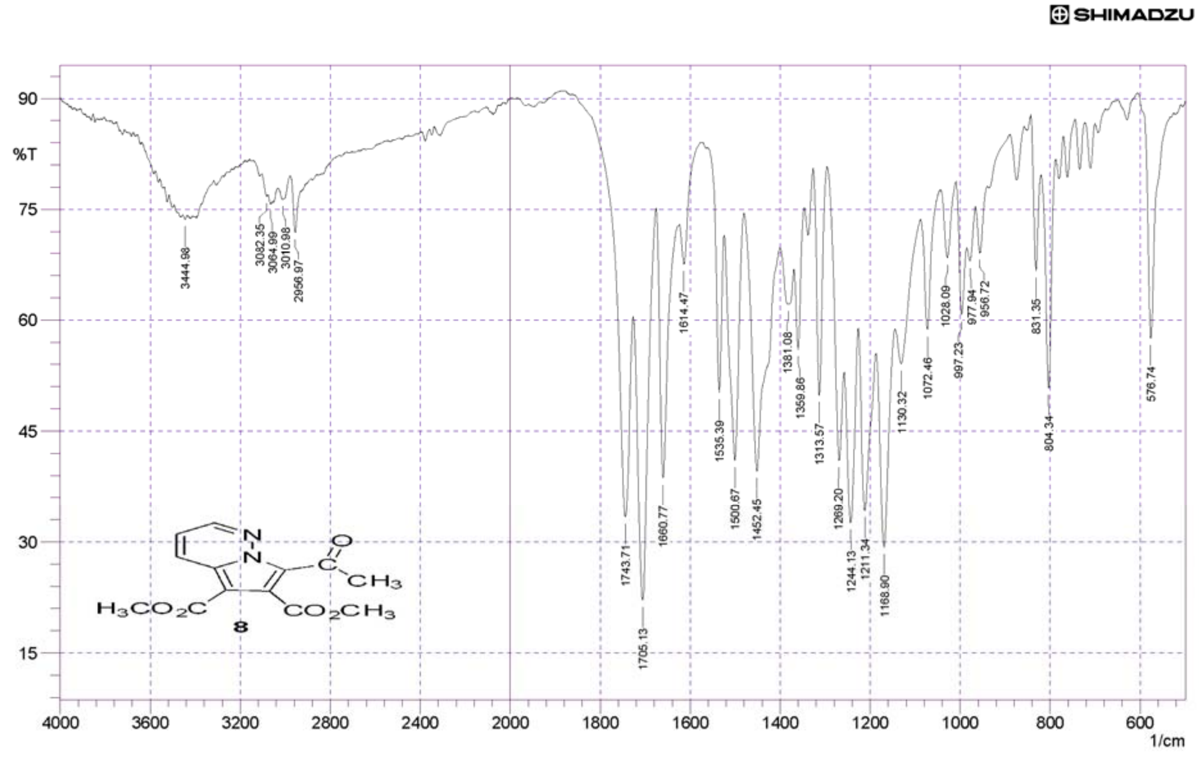

S15 Fig. IR spectrum of the compound **8.**

SHIMADZU

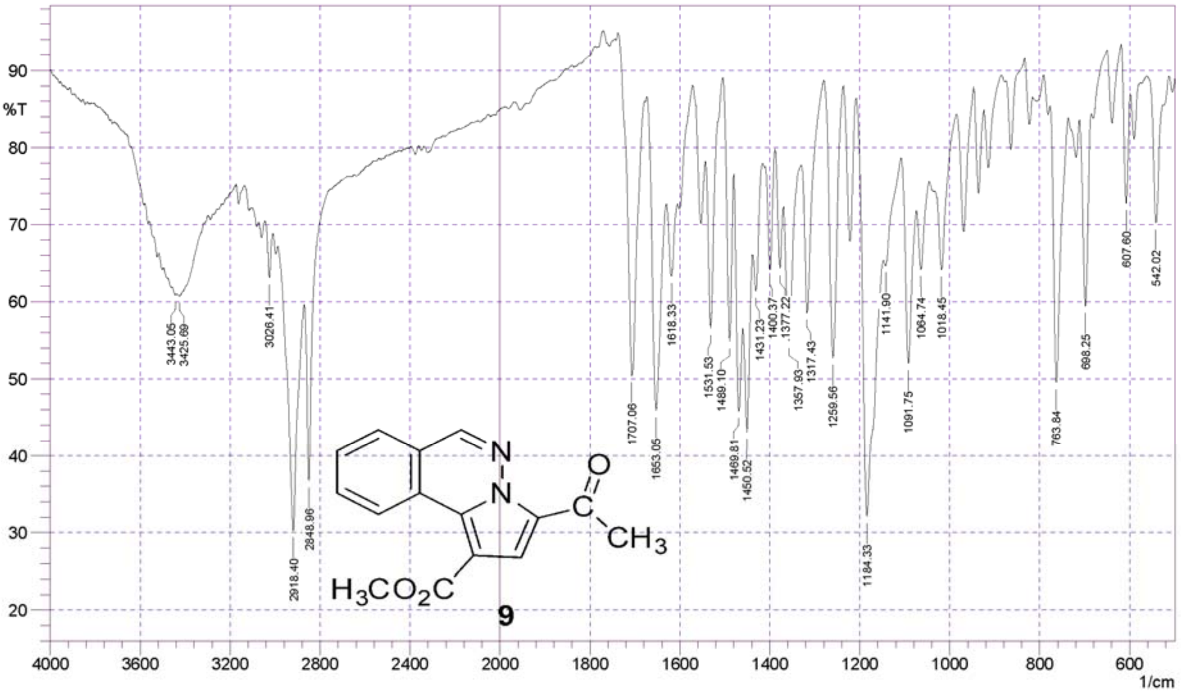

S16 Fig. IR spectrum of the compound **9.**

SHIMADZU

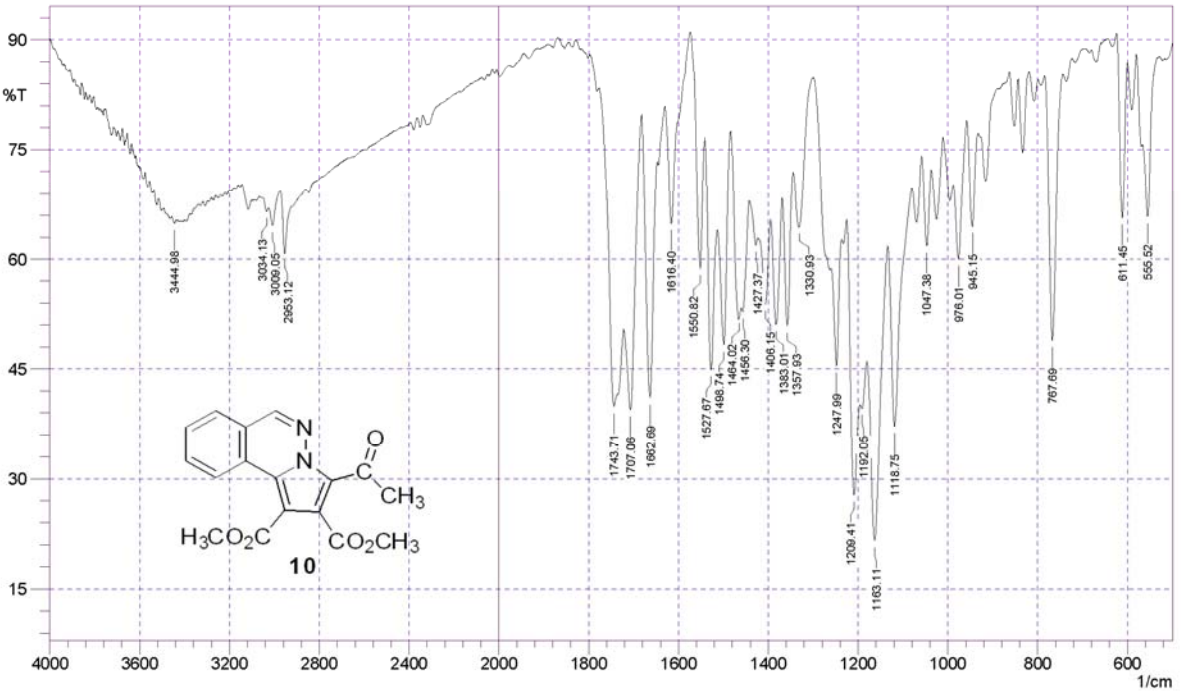

S17 Fig. IR spectrum of the compound **10.**

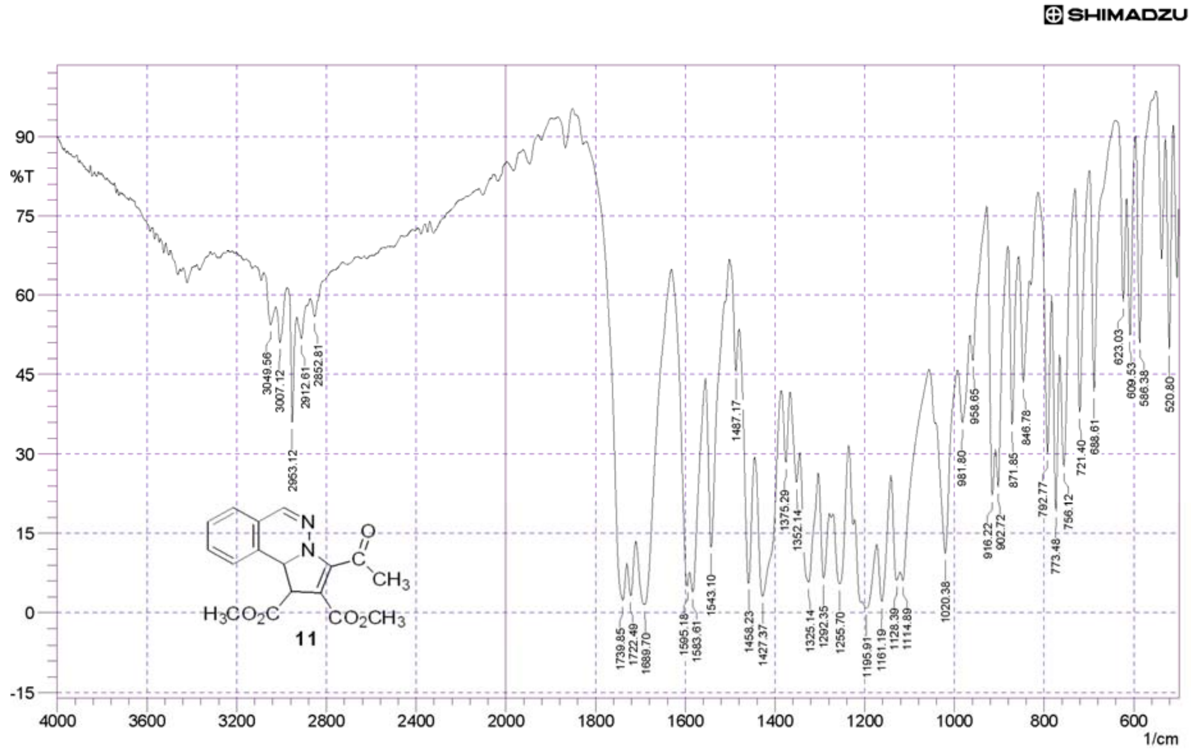

S18 Fig. IR spectrum of the compound **11.**

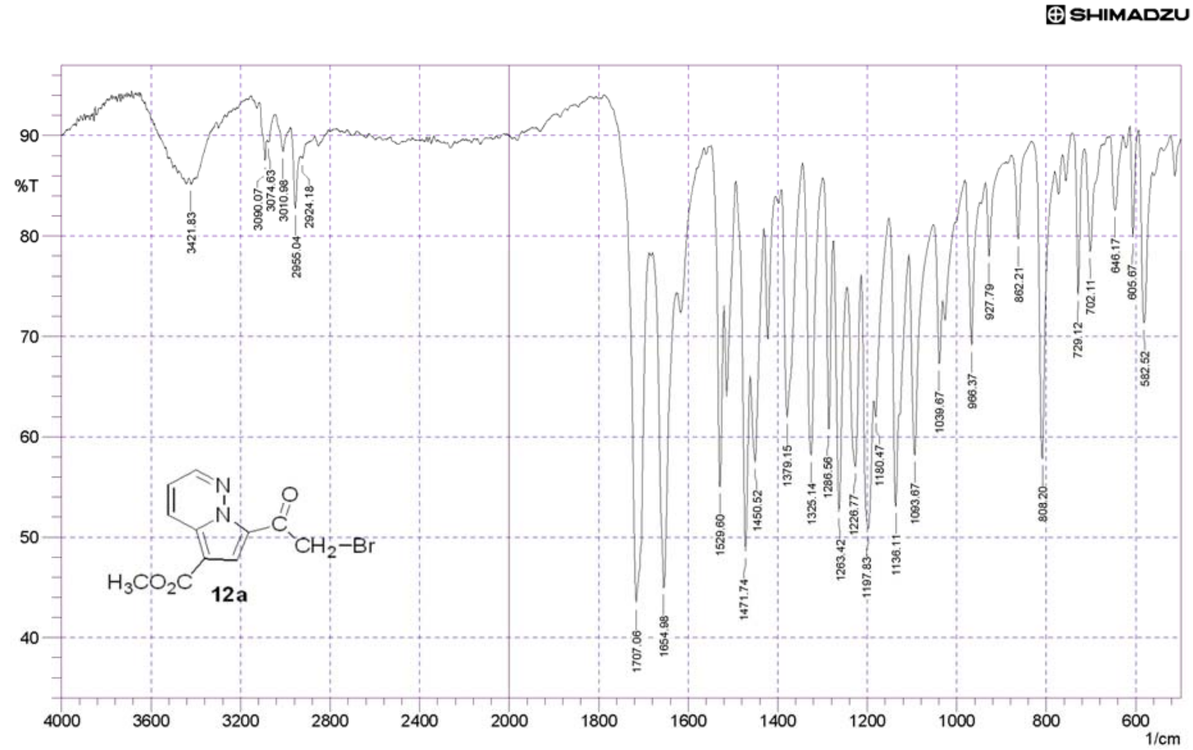

S19 Fig. IR spectrum of the compound **12a.**

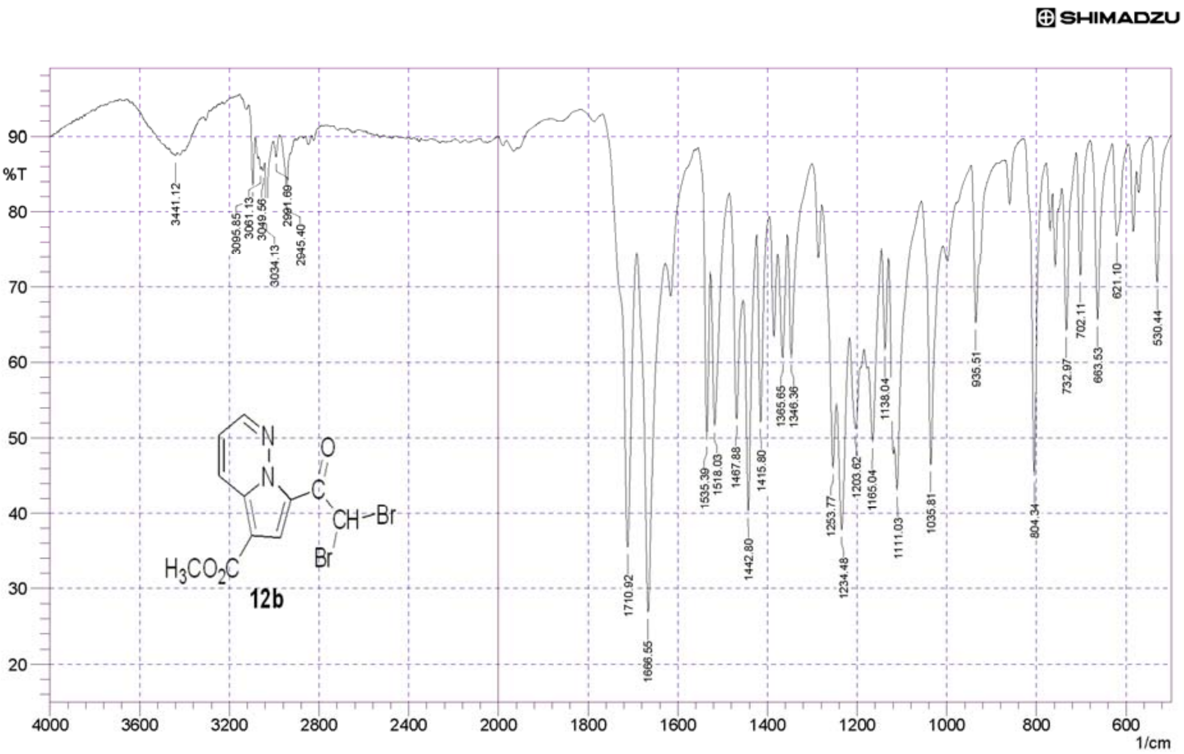

S20 Fig. IR spectrum of the compound **12b.**

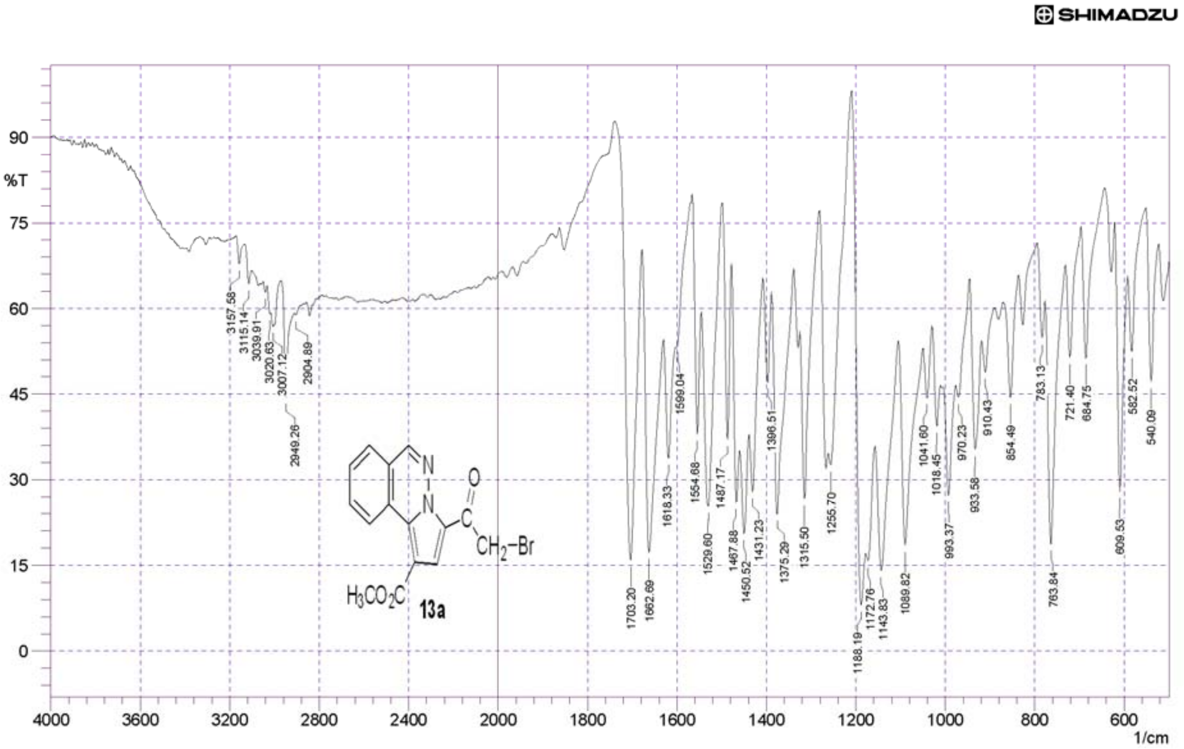

S21 Fig. IR spectrum of the compound **13a.**

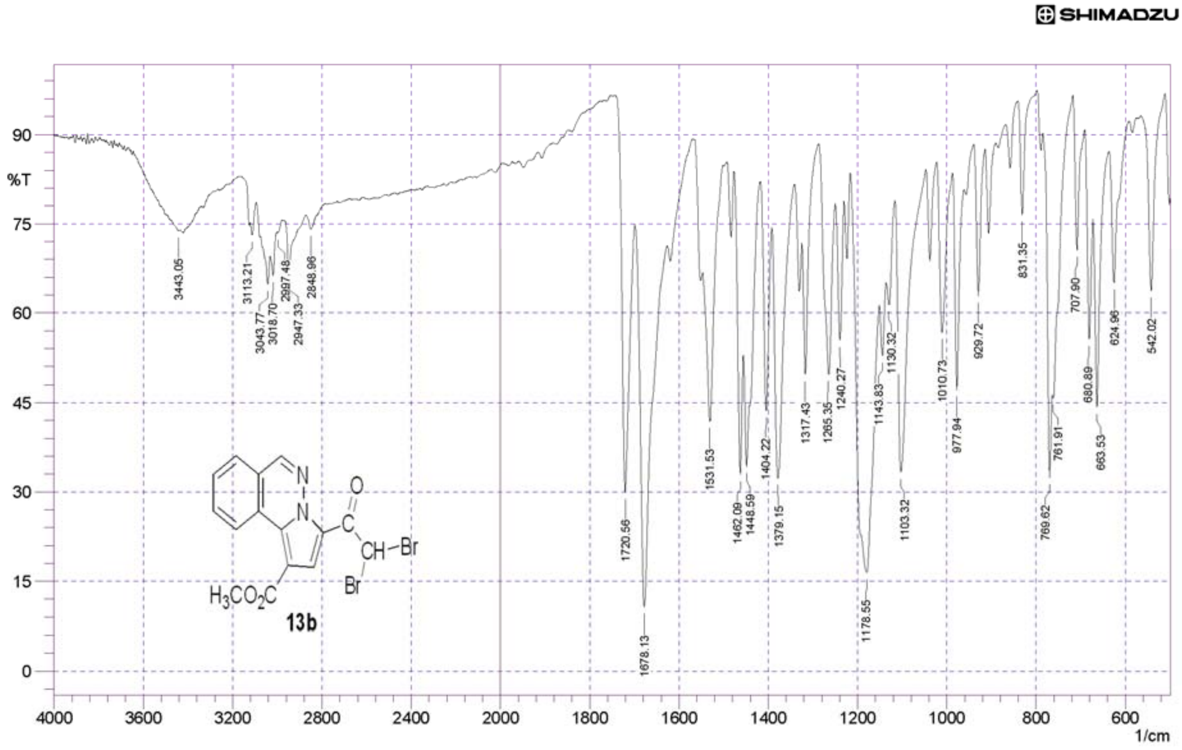

S22 Fig. IR spectrum of the compound **13b.**

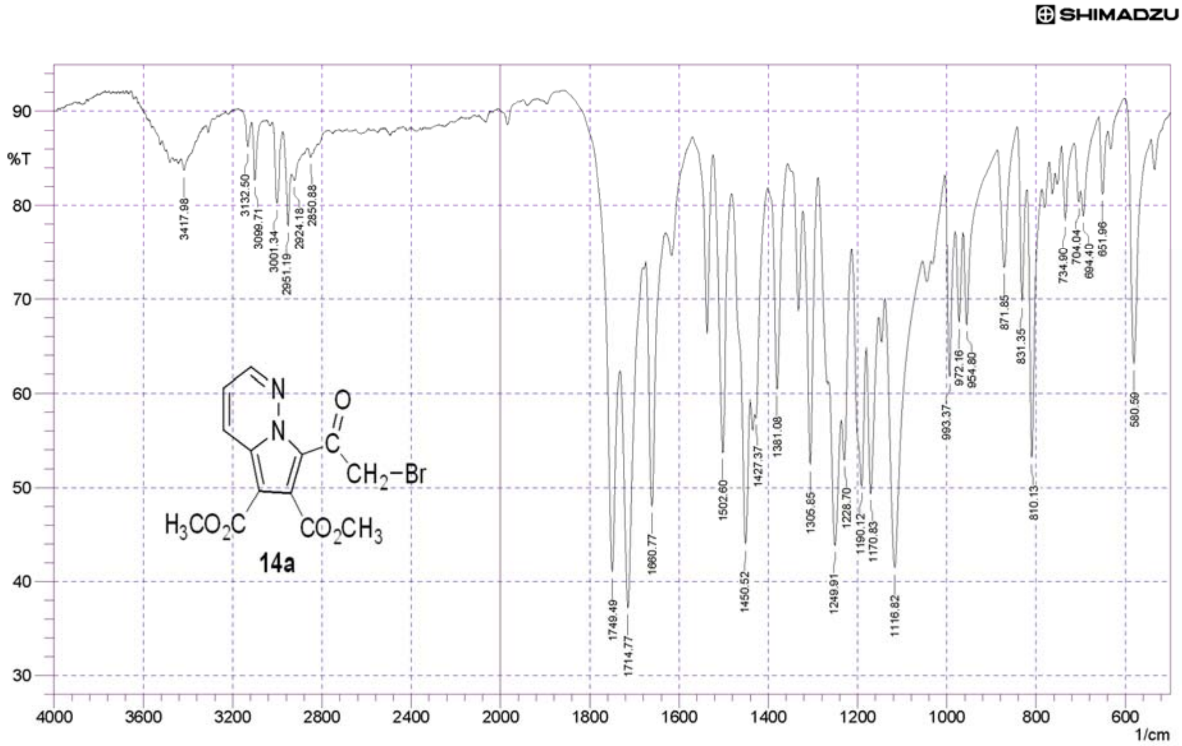

S23 Fig. IR spectrum of the compound **14a.**

SHIMADZU

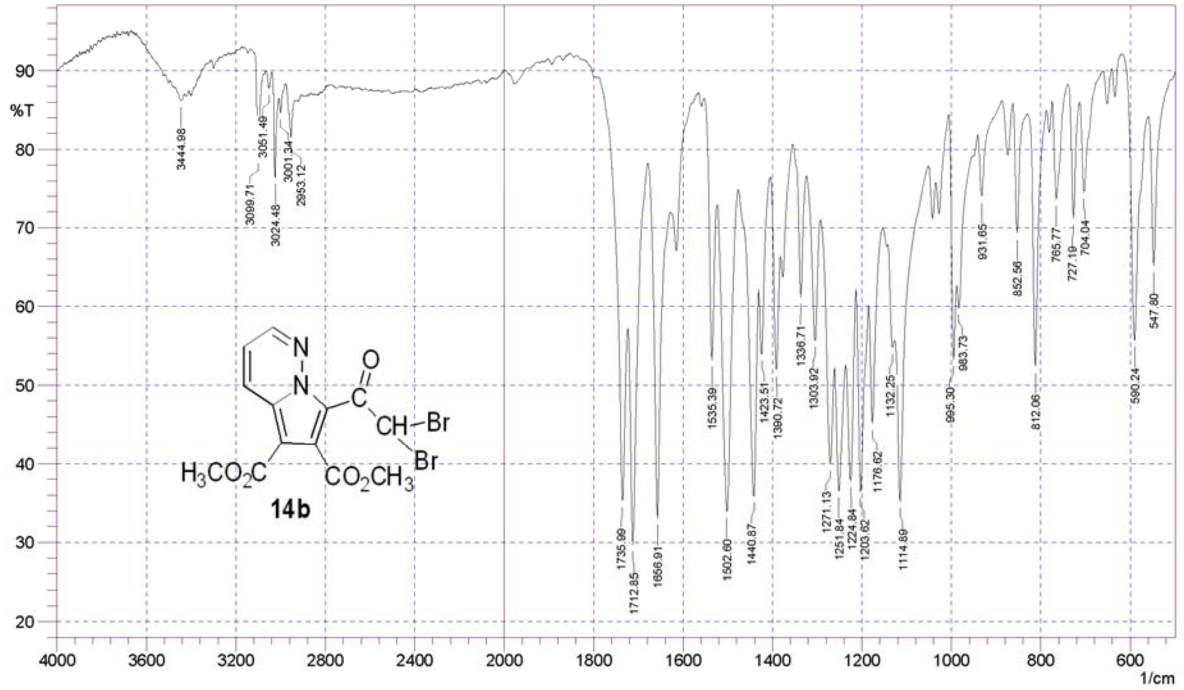

S24 Fig. IR spectrum of the compound **14b.**

SHIMADZU

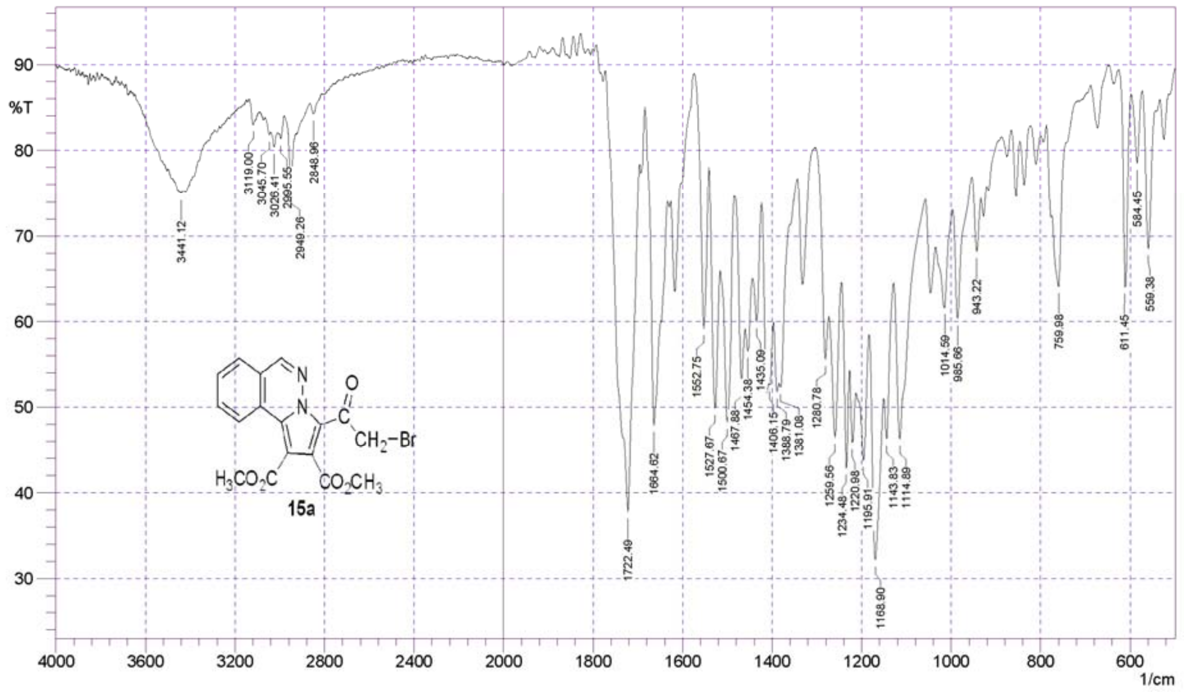

S25 Fig. IR spectrum of the compound **15a.**

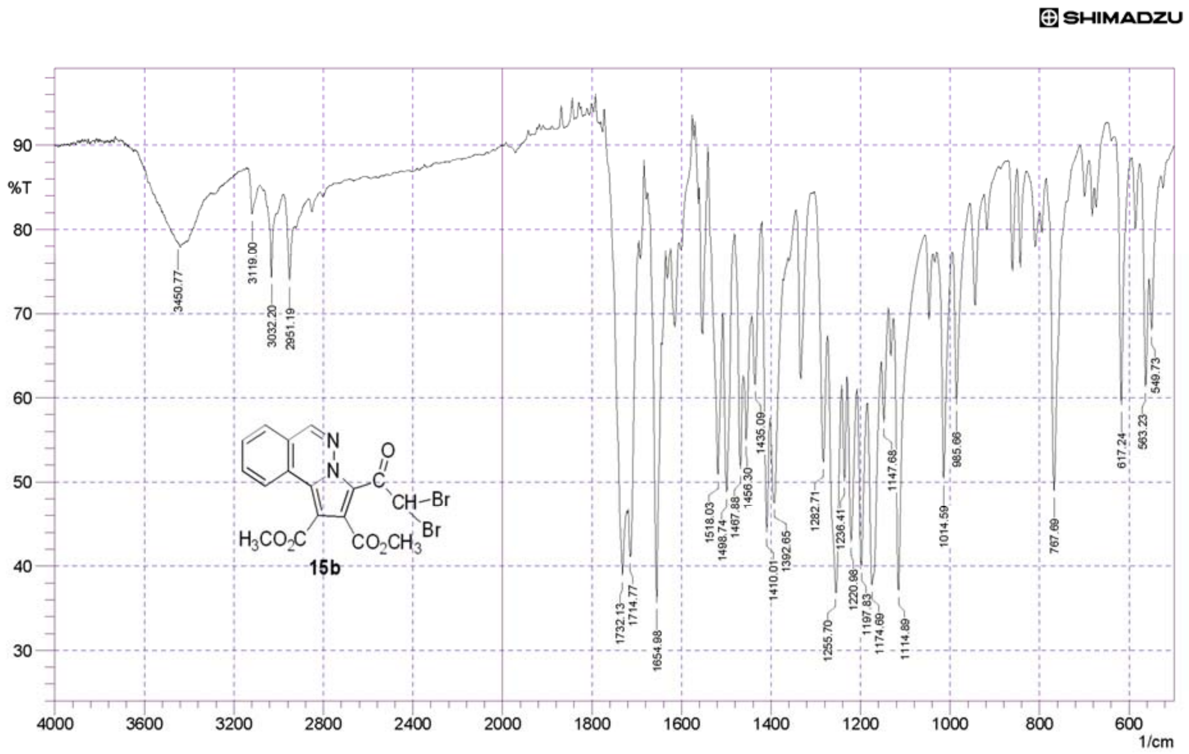

S26 Fig. IR spectrum of the compound **15b.**
